# Supplementary material for: Professionalism and Ethics: A Standardized Patient Observed Standardized Clinical Examination to Assess ACGME Pediatric Professionalism Milestones
Source: MedEdPORTAL. 2020 Jan 31;16:10873. doi: 10.15766/mep_2374-8265.10873 (PMC7062544; doi:10.15766/mep_2374-8265.10873)
Supplement: Supplementary file 1 — A. SP Case Development Tool Drug Screening.docx B. SP Case Development Tool Asthma.docx C. SP Case Development Tool Transfusion.docx D. SP Case Development Tool Mitochondrial.docx E. Door Notes.docx F. Learner Assessment Sheets.docx G. Debriefing Talking Points.docx H. Logistical Grid.docx I. Scenario Evaluations.docx J. OSCE Evaluation.docx K. Preevaluation for Preceptors.docx L. Postevaluation for Preceptors.docx [file mep-16-10873-s001.zip › _Educational_Summary_Report_10873.pdf]

MedEdPORTAL® The AAMC Journal of Teaching and Learning Resources

Original Publication Open Access

Professionalism and Ethics: A Standardized Patient Observed Standardized Clinical Examination to Assess ACGME Pediatric Professionalism Milestones

Margaret Waltz, PhD*, Arlene Davis, JD, R. Jean Cadigan, PhD, Rohit Jaswaney, Melissa Smith, MD, Benny Joyner, MD, MPH

*Corresponding author: margaret_waltz@med.unc.edu

Abstract

Introduction: The ethical skills fundamental to medical practice encompass a large portion of the Accreditation Council for Graduate Medical Education (ACGME) professionalism milestones. Yet many ethical practices are difficult to reduce to milestone frameworks given the variety of traditions of moral reasoning that clinician-trainees and their colleagues might properly employ. **Methods:** We developed an observed standardized clinical examination (OSCE) simulation with standardized patients to assess the ethical skills captured in professionalism milestones in pediatrics. The OSCE included four vignettes based on actual cases that presented problems without a correct answer. Residents discussed ethically challenging issues with standardized patients and were evaluated on specific ethical tenets contained in the professionalism milestones. Our assessment guide for preceptors offered content for debriefing and assessment. We piloted this OSCE with seven preceptors and 17 pediatric residents in two different medical settings. **Results:** Residents all agreed that the four cases were realistic. All but two residents agreed that OSCEs like this one are an appropriate or objective way of assessing the ACGME professionalism milestones. All preceptors reported that they strongly agreed the assessment improved their ability to assess the professionalism milestones. **Discussion:** This OSCE offers a structured method to assess professionalism milestones and a forum to discuss ethical problem solving. It can also be used solely as a training exercise in ethical decision making and having difficult conversations.

Keywords
Ethics, Communication, Standardized Patients, Simulation, Clinical Skills Assessment, OSCE, Pediatrics

Educational Objectives

By the end of this activity, the learner will be able to:

1. Practice navigating ethically difficult encounters and enacting professionalism skills.
2. Apply ethical reasoning to arrive at an ethically permissible course of action.

Introduction

In 1999, when the Accreditation Council for Graduate Medical Education (ACGME) and the American Board of Medical Specialties approved the six general competencies for physicians, the ethical skills fundamental to medical practice were prominently featured in professionalism milestones. As the milestones were further developed and implemented, their articulation and evaluation grew more complex.[1] Although many kinds of competencies may be hard to reduce to milestone frameworks, ethical practices are particularly problematic given the variety of traditions of moral reasoning that clinician-trainees and their colleagues might properly employ. Reliance on gut instinct in competency evaluation is perhaps especially problematic in the development of ethical practice, as it distracts from the rigor of established ethics methods central to professional practice.[2] In 2012, Cook, Sobotka, and Ross[3] surveyed pediatric program directors regarding ethics and professionalism practices in their programs and found that most programs lacked rigorous evaluation of trainee competency in ethics and professionalism. The ACGME has yet to provide a definitive method for mentoring professionalism. As a result, few residency programs have established curricula in professionalism, with many programs expressing a desire for improvement in evaluation measures.[4,5] The lack of structured curricula and evaluation tools highlights the need for such a program.[5]

Citation:
Waltz M, Davis A, Cadigan RJ, Jaswaney R, Smith M, Joyner B. Professionalism and ethics: a standardized patient observed standardized clinical examination to assess ACGME pediatric professionalism milestones. *MedEdPORTAL*. 2020;16:10873.
https://doi.org/10.15766/mep_2374-8265.10873

Copyright © 2019 Waltz et al. This is an open-access publication distributed under the terms of the Creative Commons Attribution license.

Traditionally, a trainee's ethics competency has been assessed via direct observations. Feedback can be sporadic, or overlooked, due to clinical demands and time constraints.[6] Although the use of an observed standardized clinical examination (OSCE), via simulation, is a well-developed tool in other parts of medical education,[7-13] there are limited data on the use of OSCEs for assessing ethical skills and professionalism at the resident level. Given the adaptability of the OSCE model and its growing prominence as an important educational tool, members of this research team familiar with simulation recognized its potential use in situations where the clinical skills assessed were based in moral reasoning and communication. Other team members, who routinely taught residents about professionalism and moral reasoning in didactic and case-based discussions, also viewed the simulation as offering an appealing venue to assess variation in approaches to dilemmas.

Recognizing that these OSCEs could showcase moral reasoning and variation in approach in a nonthreatening and low-stakes environment, we chose this approach for the project to provide a necessary formalized training of professionalism and to inform its practical application. Therefore, we developed an OSCE composed of four 15-minute simulations with standardized patients to assess the ethical skills captured in pediatric professionalism milestones.

This OSCE project grew out of another study our team conducted analyzing the types of ethical dilemmas that pediatric residents found particularly difficult.[14] That study used observations over 10 months of a long-standing ethics education series for residents. In these education sessions, residents nominated for discussion the cases from their clinical practice they found challenging. Our observation study documented these cases and the ensuing discussions, which resulted in an inventory of ethics cases. The four simulations for this OSCE were developed from some of these cases.

The multidisciplinary nature of our research team, including supervising physicians with simulation center experience, a medical student trainee, a lawyer ethicist, and social scientists, was an essential feature of this project. Most of the team members are also members of our institution's Clinical Ethics Service and Hospital Ethics Committee. Using the inventory of ethics cases, our team met to discern which cases were the most relevant to clinical practice, seeking cases that had a diversity of clinical settings, severity of illnesses, and ages of patients. We then narrowed the possible cases based on how actionable they were for simulation in terms of how long a case would take to act out or how many actors would be needed for it. Of the cases that remained, we then discussed which ones aligned with professionalism milestones. Milestones were assigned by highlighting several ethical considerations that residents could potentially approach when simulating the case. Through an iterative process, we tailored the cases to specific ACGME milestones in professionalism, specifically, milestones 1, 2, 5, and 6. Using existing models of assessment sheets from the University of North Carolina at Chapel Hill School of Medicine's Simulation Center, we developed an assessment sheet around the primary milestone for each case. These assessment sheets were developed to highlight the residents' ability to professionally navigate the simulation without requiring them to approach the case in one correct way. We reviewed the assessment sheets as a team, shared them with other clinicians for refinement, and made minor revisions after initial resident assessments.

Our OSCE method differs from the Association of Pediatric Program Directors/American Board of Pediatrics (APPD/ABP) curriculum[15] in that the simulations described provide four discrete scenarios with accompanying evaluation rubrics and structure for designing and executing scenarios. Although not intended to be an all-encompassing ethics education assessment, this curriculum provides concrete, step-by-step instructions on setup and execution of simulations around four situations typically encountered in an academic medical center that have been described as particularly distressing or difficult by recent trainees. This assessment also differs from the APPD/ABP curriculum in that it gives residents the chance to experience ethical clinical scenarios with targeted feedback directly linked to the required ACGME milestone objectives in a setting where residents can perform without concern about how the patient or family will react. Specific feedback regarding managing difficult conversations, ethical decision making, and partnering with parents can be discussed in the allotted time immediately after the simulation has completed. This gives the advantage of direct, immediate feedback to an observed situation. Similar studies have been performed with the intent to find an objective, reproducible manner to evaluate communication and professionalism across training programs or interdisciplinary teams.[16] Our program differs by sampling residents across any level of training and attempting to comment on the growth of professionalism throughout the residency program.

Methods

The target audience for this OSCE included general pediatric residents at any level of training.

Copyright © 2019 Waltz et al. This is an open-access publication distributed under the terms of the Creative Commons Attribution license.

Logistics

Residents spent 1 hour completing this OSCE of four simulations. Each simulation lasted 15 minutes, with about 10 minutes spent with the standardized patient (including reading the door note with the scenario description provided in Appendix E) and 5 minutes of debriefing with the preceptor using the assessment sheet (Appendix F) and debriefing notes (Appendix G). Although we ran the assessment by having residents complete all four simulations back to back, the simulations need not be performed together. The number of cases completed at one time could vary based on time available for residents and preceptors. More time could also be provided for feedback.

If performing all four cases together, Appendix H presents a grid for how residents should move through the assessment so that four residents can participate at one time. Residents were given their portion of the grid as their schedule. It was easiest to color code the scenarios to clearly indicate to residents the order in which they would be completing the simulations. The scripts for the standardized patients, the assessment sheets, and the debriefing materials for the preceptors were also color coded for ease of distribution. It was also important to bring extra copies of everything, particularly the door notes, which were often carried off by residents.

Environment

Four rooms were necessary for the simulations, one for each individual scenario. Each room had a color-coded door note on the door for residents to read before entering the room. Residents completed the simulation with the standardized patient and the debriefing with the preceptor in the same room. The rooms did not need anything more than space for three individuals (the resident, standardized patient, and preceptor) and chairs for the resident and standardized patient. The preceptor stood behind or out of sight of the resident.

Personnel

The University of North Carolina at Chapel Hill School of Medicine Simulation Center has a pool of standardized patients. We used the Simulation Center's services to reserve rooms and hire standardized patients when conducting the simulations at the university. We also ran the OSCEs at an off-site hospital and used small conference rooms and our own team members as standardized patients. Therefore, using Simulation Center resources was not required. When running the simulations, four standardized patients were necessary: One simulation involved an adolescent patient, whereas the other three involved parents. The gender, age, and other characteristics of patients and parents/caregivers in the scenarios could change based on who was available to play the standardized patient or for educational purposes. Four medical school physician faculty or other experienced preceptors were necessary to serve as preceptors. One person was also needed to train the standardized patients. Finally, it was helpful to have a facilitator who answered any questions residents had as they moved through the assessment.

Preparation

Before the simulations began, standardized patients reviewed the door notes for their cases with the instructions for standardized patients (Appendix E) and their standardized patient case (Appendices A-D), and they received specific training regarding the purpose of the assessment. Prior to the onset of the case, a trainer met with the standardized patient to discuss the case in detail, identify the objective in the case, review potential answers to the questions involved, and discuss expected parental and patient interactions and concerns. Given that each case was targeted to a very specific situation (and involved pediatric patients), only the social history was discussed in detail. The general medical history was kept as simple as possible to avoid confusion or conflicting responses. Areas of concern or areas in which there could be misinterpretations were discussed prior to the simulation. Specific responses were not scripted; however, each standardized patient was provided the context for each response and the wide range of responses typically encountered so that if a resident did not respond as expected, the standardized patient would have an understanding of an appropriate response. The standardized patients were given an opportunity to rehearse their respective roles prior to beginning the assessment. This training took about 30 minutes, during which the trainer also answered any questions the standardized patients had about how to play the role. Using the same standardized patients for any subsequent sessions would, of course, reduce the time necessary for training. The instructions for the standardized patients could be edited to make the scenarios more or less challenging for residents, particularly by instructing the standardized patients on the level of conflict they should portray during the simulation. Each simulation introduced points of potential opposition within its context.

The training of preceptors was similarly performed by discussing expected responses for each event. For their assigned case, preceptors reviewed the scenario door note, assessment sheet, and debriefing materials and were trained on using the learner assessment sheet (see below and Appendix F). Each preceptor

Copyright © 2019 Waltz et al. This is an open-access publication distributed under the terms of the Creative Commons Attribution license.

was instructed to fill out the yes/no column of the assessment sheet based on his or her observation of the behavior described. If the preceptor checked no, he or she was to elaborate on why the behavior was not met in the comments section. If the resident only partially enacted the behavior, the preceptor was to check the no box and use the comments section to provide further explanation. Preceptors were assigned to observe one specific case in order to become familiar with it and to see nuances across the residents' performances.

Learner Assessment

Preceptors used the assessment sheets and debriefing notes to provide feedback to the resident after the simulation was completed. The assessment sheet featured a checklist to determine whether the resident upheld the professionalism milestones while also allowing for multiple paths to be taken during the simulation, as there was no one right way to complete the simulations. The debriefing guide helped the preceptor discuss the resident's ethical problem solving. The assessments were intended to be used by physicians for physician learners, not for peer-to-peer evaluation.

Results

To date, we have run the assessment six times with 17 pediatric residents. Seven people have served as preceptors, three of whom (Benny Joyner, Melissa Smith, and Arlene Davis) are members of our study team.

For our study purposes, residents completed evaluations of all four individual scenarios (Appendix I) and the overall OSCE (Appendix J). Residents appreciated the OSCE, all agreeing that the four cases were realistic. We asked residents what they perceived to be the most useful part of the OSCE. From their responses, we identified three common themes. First, nine residents noted the benefits of practicing various aspects of difficult encounters, including having difficult conversations with patients and parents, ethical decision making, and partnering with parents. Representative comments included that the OSCE was "a great test of how we might actually respond," served "as a reminder to be mindful of the way we speak with patients and families," provided an "opportunity to practice partnering with parents," and was "helpful to be able to practice tough positions and conversations before having to have them with families." Second, five residents said the ability to get "concrete feedback" from attendings was useful. Finally, three residents stated the assessment was useful because the cases were realistic. Two of these residents said the simulations were useful because "they were scenarios that occur," whereas one noted their utility because "some of these scenarios are things we do not always get to experience that frequently."

The transfusion case (Appendix C) stood out to the majority of residents (10 of 17) as the case that best assessed professionalism because of the emotion and empathy required to address the situation (three residents), the difficult conversation required (two residents), the complexity of the case that required multiple teams (two residents), and the struggle to balance professional duty with a parent's religious beliefs (two residents). Interestingly, 11 of 17 residents also said this case was the most challenging. For the remaining residents, similar themes emerged regarding how well the other cases assessed professionalism, including the benefit of practicing difficult conversations and developing action plans.

Attendings and fellows who served as preceptors (and who were not part of the study team) were given a pre- and post-OSCE evaluation (Appendices K and L). In the pre-OSCE evaluation, preceptors were asked how comfortable they were giving residents feedback regarding the ethical issues that appear in the professionalism milestones (5-point scale: 1 = *not at all comfortable*, 5 = *very comfortable*). No one reported being very comfortable providing such feedback. As one preceptor said, professionalism was "hard to teach someone in terms of setting people up with the tools they need . . . . Lots of conversations with patients and families for trainees happen on their own. They are hard to see." Another stated it was difficult to evaluate due to time: "There are so many things we have to do." In the post-OSCE evaluation, all preceptors reported on a 5-point scale (1 = *strongly disagree*, 5 = *strongly agree*) that they strongly agreed the OSCE improved their ability to assess the professionalism milestones. All four preceptors valued the ability to observe residents of varying experience levels and tailor the feedback to each person's needs. They all also commented on how they liked that the OSCE provided time to give direct and immediate feedback on difficult patient interactions.

Discussion

We conducted this formative assessment OSCE with pediatric residents in an effort to formalize training in professionalism and ethics. The OSCE was designed to assess ethical skills associated with ACGME professionalism milestones. The individual simulations were observed by preceptors who were given talking points for debriefing discussions. The simulations provided a structured opportunity for preceptors to view how different residents approached the same situation and allowed

Copyright © 2019 Waltz et al. This is an open-access publication distributed under the terms of the Creative Commons Attribution license.

residents to practice their ethical reasoning skills and receive real-time feedback about the ethical dilemmas contained in the simulations. Importantly, the assessment provided time for preceptors and residents to discuss ethical decision making, which can often be lacking in resident training.[17]

The OSCE can be used with junior and senior pediatric residents at any level of ethical reasoning training and professionalism skills development. The scenarios are designed to provide decision points to residents. The assessment sheets allow residents to take different paths during the simulations, and the discussion guide provides preceptors tips on how to debrief with the residents about the decisions that have been made.

The four cases can be adapted for specific institutional needs. The cases can be tailored to include local policy and law relevant to any training program. They can also be adapted to highlight and assess other professionalism milestones depending on how institutions decide to focus their resident evaluations. Standardized patients can be coached to give more or less pushback to residents at certain points in the scenarios. Additionally, the ages of some of the patients can change to young adult status to bring out issues of assent. Finally, the severity of patient illness can also be adjusted, which arguably influences how challenging residents perceive the case to be, as evidenced by the transfusion case being rated the most difficult by residents in this study.

Limitations to this study include the small sample size of preceptors and residents, which restricts the generalizability of the study. Preceptors were in the room with residents, which allowed them to provide immediate feedback to the residents, but that may have impacted residents' comfort and performance. However, if there is not an option for preceptors to observe the simulations in real time through a window or camera, it may be necessary for preceptors to be in the room during the simulation. We also had limited assessments of the impact of the OSCE, focusing mostly on learner perceptions. However, learners and preceptors both appreciated the opportunity for real-time feedback on challenging ethical dilemmas, which we believe is a valuable contribution of this assessment. Future iterations of this study would benefit from changes to the evaluation forms completed by preceptors and residents following assessment. For the preceptor form, adding questions about comfort in assessing each individual milestone would allow for direct comparison to the preevaluation form. Additionally, the postevaluation form for residents would be improved by asking residents to comment on the effectiveness of the debriefing sessions as an educational tool. The form could also ask residents to comment on their perceptions of the utility of the OSCEs for assessing professionalism as compared with their other experiences of assessment.

Challenges to running the OSCE include preceptors and residents finding time in their busy schedules to attend the assessment and complete the evaluations. This challenge comes with the benefit of providing time for residents and preceptors to have discussions about ethics and professionalism. As noted, the four cases do not all have to be completed at once, which can provide more time for feedback. However, time constraints limited the number of survey questions we could ask of residents and preceptors, which led to limitations of our study described above. Additionally, there is a challenge of having resources for simulations, especially space, money for standardized patients, and the ability to recruit age-appropriate standardized patients. Also needed are personnel able to train the standardized patients following best-practice guidelines.[18] As already noted, the OSCE can be run without professional standardized patients in rooms with just a few chairs. We observed no differences in the educational experience of residents who completed the assessment in a simulation center and those who completed it in an office setting. The standardized patients who were not professionals were trained in the same way as the professional standardized patients prior to the assessment. However, personnel familiar with running simulations are necessary to create the same type of experience at sites without simulation centers.

In conclusion, this assessment provides a structured way to assess professionalism milestones and have a forum to discuss ethical problem solving in real time. The cases permit flexibility and can also be used solely as training exercises in ethical decision making and communication skills, even if not used to assess the ACGME professionalism milestones.

Appendices

A. SP Case Development Tool Drug Screening.docx
B. SP Case Development Tool Asthma.docx
C. SP Case Development Tool Transfusion.docx
D. SP Case Development Tool Mitochondrial.docx
E. Door Notes.docx
F. Learner Assessment Sheets.docx
G. Debriefing Talking Points.docx

Copyright © 2019 Waltz et al. This is an open-access publication distributed under the terms of the Creative Commons Attribution license.

H. Logistical Grid.docx
I. Scenario Evaluations.docx
J. OSCE Evaluation.docx
K. Preevaluation for Preceptors.docx
L. Postevaluation for Preceptors.docx

All appendices are peer reviewed as integral parts of the Original Publication.

Margaret Waltz, PhD: Research Associate, Department of Social Medicine, University of North Carolina at Chapel Hill School of Medicine

Arlene Davis, JD: Associate Professor, Department of Social Medicine, University of North Carolina at Chapel Hill School of Medicine; Associate Professor, Center for Bioethics, University of North Carolina at Chapel Hill

R. Jean Cadigan, PhD: Associate Professor, Department of Social Medicine, University of North Carolina at Chapel Hill School of Medicine; Associate Professor, Center for Bioethics, University of North Carolina at Chapel Hill

Rohit Jaswaney: Medical Student, New York Medical College

Melissa Smith, MD: Assistant Professor, Department of Pediatrics, University of North Carolina at Chapel Hill School of Medicine; Assistant Professor, Department of Anesthesia, University of North Carolina at Chapel Hill School of Medicine

Benny Joyner, MD, MPH: Associate Professor, Department of Pediatrics, University of North Carolina at Chapel Hill School of Medicine; Associate Professor, Department of Anesthesia, University of North Carolina at Chapel Hill School of Medicine; Associate Professor, Department of Social Medicine, University of North Carolina at Chapel Hill School of Medicine

Acknowledgments
We would like to thank Warren Whipple for helpful comments and assistance in developing this study. Many thanks also to those who participated in the study.

Disclosures
None to report.

Funding/Support
Support for this publication was provided by an AHEC Campus Innovation Grant to Arlene Davis, principal investigator.

Prior Presentations
Waltz M, Davis A, Cadigan RJ, Smith M, Jaswaney R, Joyner B. We know it when we see it: reducing subjectivity in the assessment of professionalism milestones. Presented at: Academy for Professionalism in Health Care Annual Meeting; May 15-17, 2019; New Orleans, LA.

Ethical Approval
The Institutional Review Board at the University of North Carolina at Chapel Hill approved this study.

References

1. Hicks PJ, Margolis M, Poynter SE, et al; for APPD LEARN-NBME Pediatrics Milestones Assessment Group. The Pediatrics Milestones Assessment Pilot: development of workplace-based assessment content, instruments, and processes. *Acad Med*. 2016;91(5):701-709. https://doi.org/10.1097/ACM.0000000000001057
2. Sklar DP. Competencies, milestones, and entrustable professional activities: what they are, what they could be. *Acad Med*. 2015;90(4):395-397. https://doi.org/10.1097/ACM.0000000000000659
3. Cook AF, Sobotka SA, Ross LF. Teaching and assessment of ethics and professionalism: a survey of pediatric program directors. *Acad Pediatr*. 2013;13(6):570-576. https://doi.org/10.1016/j.acap.2013.07.009
4. Kesselheim JC, Sectish TC, Joffe S. Education in professionalism: results from a survey of pediatric residency program directors. *J Grad Med Educ*. 2012;4(1):101-105. https://doi.org/10.4300/JGME-D-11-00110.1
5. Lang CW, Smith PJ, Ross LF. Ethics and professionalism in the pediatric curriculum: a survey of pediatric program directors. *Pediatrics*. 2009;124(4):1143-1151. https://doi.org/10.1542/peds.2009-0658
6. Chen HC, van den Broek WES, ten Cate O. The case for use of entrustable professional activities in undergraduate medical education. *Acad Med*. 2015;90(4):431-436. https://doi.org/10.1097/ACM.0000000000000586
7. Antoun J, Romani M, Saab B. Disclosure of medical error: objective structured clinical examination (OSCE). *MedEdPORTAL*. 2012;8:9226. https://doi.org/10.15766/mep_2374-8265.9226
8. Lupi CS, Ward-Peterson M, Castro C. Non-directive pregnancy options counseling: online instructional module, objective structured clinical exam, and rater and standardized patient training materials. *MedEdPORTAL*. 2017;13:10566. https://doi.org/10.15766/mep_2374-8265.10566
9. Ben-Isaac E, Zipkin R, Thompson M. Modified objective structured clinical examinations for PL-3 pediatric residents. *MedEdPORTAL*. 2016;12:10382. https://doi.org/10.15766/mep_2374-8265.10382
10. Ludwig A, Lee R, Parish S, Raff A. Four-station group observed structured clinical encounter for formative assessment of communication skills for internal medicine clerks. *MedEdPORTAL*. 2016;12:10444. https://doi.org/10.15766/mep_2374-8265.10444

Copyright © 2019 Waltz et al. This is an open-access publication distributed under the terms of the Creative Commons Attribution license.

11. Lamba S, Bryczkowski S, Holland B, Nagurka R, Mosenthal A. A teaching OSCE to assess trauma resident skills on how to hold difficult conversations with family of critically injured patients. *MedEdPORTAL*. 2016;12:10398. https://doi.org/10.15766/mep_2374-8265.10398

12. Aeder L, Altshuler L, Kachur E, Langenau E. Fostering an atmosphere of professionalism in a residency program: learning how to address the unprofessional behavior of colleagues through OSCEs. *MedEdPORTAL*. 2012;8:9168. https://doi.org/10.15766/mep_2374-8265.9168

13. Willis DR, Renshaw S, Pais S, Custer J, Krebs EE, Kiovsky R. Preventative care objective structured clinical examination (OSCE) based on USPSTF guidelines December 2011. *MedEdPORTAL*. 2012;8:9185. https://doi.org/10.15766/mep_2374-8265.9185

14. Waltz M, Cadigan RJ, Joyner B, Ossman P, Davis A. Perils of the hidden curriculum: emotional labor and "bad" pediatric proxies. *J Clin Ethics*. 2019;30(2):154-162.

15. American Board of Pediatrics Education and Training Committee. *Teaching, Promoting and Assessing Professionalism Across the Continuum: A Medical Educator's Guide*. 2nd ed. American Board of Pediatrics website. https://www.abp.org/professionalism-guide. Accessed August 2, 2019.

16. Gardner AK, Lim G, Minard CG, Guffey D, Pillow MT. A cross-specialty examination of resident error disclosure and communication skills using simulation. *J Grad Med Educ*. 2018;10(4):438-441. https://doi.org/10.4300/JGME-D-17-00603.1

17. Deonandan R, Khan H. Ethics education for pediatric residents: a review of the literature. *Can Med Educ J*. 2015;6(1):e61-e67.

18. Lewis KL, Bohnert CA, Gammon WL, et al. The Association of Standardized Patient Educators (ASPE) Standards of Best Practice (SOBP). *Adv Simul (Lond)*. 2017;2:10. https://doi.org/10.1186/s41077-017-0043-4

Received: March 19, 2019
Accepted: September 11, 2019
Published: January 31, 2020

Copyright © 2019 Waltz et al. This is an open-access publication distributed under the terms of the Creative Commons Attribution license.
