# Supplementary material for: Professionalism and Ethics: A Standardized Patient Observed Standardized Clinical Examination to Assess ACGME Pediatric Professionalism Milestones
Source: MedEdPORTAL. 2020 Jan 31;16:10873. doi: 10.15766/mep_2374-8265.10873 (PMC7062544; doi:10.15766/mep_2374-8265.10873)
Supplement: Supplementary file 1 — A. SP Case Development Tool Drug Screening.docx B. SP Case Development Tool Asthma.docx C. SP Case Development Tool Transfusion.docx D. SP Case Development Tool Mitochondrial.docx E. Door Notes.docx F. Learner Assessment Sheets.docx G. Debriefing Talking Points.docx H. Logistical Grid.docx I. Scenario Evaluations.docx J. OSCE Evaluation.docx K. Preevaluation for Preceptors.docx L. Postevaluation for Preceptors.docx [file mep-16-10873-s001.zip › A. SP Case Development Tool Drug Screening.docx]

Appendix A: *MedEdPORTAL* Standardized Patient Case Development Tool

Date: 5/9/19

Primary Case Author: Margaret Waltz, Arlene Davis, R. Jean Cadigan

Secondary Case Author: Rohit Jaswaney, Melissa Smith, Benny Joyner

Standardized Patient Educator: Melissa Smith and Benny Joyner

Name of Case: Drug Screening in the Clinic

Name of educational and or assessment activity: Professionalism and Ethics Simulation

Patient Name: Emily

Chief Complaint: Patient needs an ADHD medical refill

Most likely Diagnosis and Differential with rationale from history and/or physical exam: The scenario is not about getting the correct diagnosis, but the decision process and the conversation with the patient.

Challenge question: Respond to the patient’s concerns about the drug screening and make a plan with her for next steps.

Domains: Check all that apply

X Professionalism

X Communication and Interpersonal skills

- Medical History
- Physical exam

X Shared Decision Making

- Patient Education
- Clinical Reasoning
- Documentation
- Handoff
- Presentation
- Other:

Type and level of learner: Pediatric residents at any level of training

Case Objectives: please list specific objectives for each of the domains you have checked above:

1. Identify at least one ethical issue related to professionalism in each case simulation

2. Apply ethical reasoning to arrive at an ethically permissible course of action

| SETTING: outpatient, in patient, ED, home, nursing home, rehab, group etc. | Outpatient |
| --- | --- |
| PATIENT PROFILE: Information about the “patient” that helps select an SP and helps the learner get an understanding of them as a person. SP will know more information about the patient than learner will ever ask but allows SP to portray a fully developed patient personality. If none of the items below are particulars for the case please write “all may be used.” | |
| Age range | 15-17 years of age |
| Religious/spiritual background | Episcopalian |
| Sex (e.g., male, female, intersex, transwoman, transman) | Female typically used; Male can be used, Michael Hendricks |
| Sexual Orientation (e.g., heterosexual, lesbian, gay, bisexual, pansexual, queer, asexual) | heterosexual |
| Gender expression (e.g., man, woman, gender queer) | Male or female depending on gender chosen |
| Race/ethnicity: | All may be used |
| Physical description (e.g., BMI, height range) | Not applicable |
| Physical limitations | None |
| Patient appearance (e.g., disheveled, hospital gown, business casual, casual) | Casual dress |
| Moulage + location (e.g., none, bruises, scars, body piercing, tattoos) | No bruises or other injuries; no evidence of drug use (i.e.-no track marks, etc) |
| Affect (e.g., pleasant, cooperative) | Nervous, evasive at first about the underlying problem and will not directly answer the question |
| Family group (e.g., who is family, who they live with) | The patient has a father who will be picking up his/her prescription |
| Education | High school student who gets As and Bs; extramural activity is cross country/track |
| Level of health literacy | Age appropriate-understands “health” but no real knowledge of drug interaction or experience with illness given that they have only come to the doctor for well-child checks and sees the pediatrician as the place to get vaccines. |
| Employment, if any - present and past, noting any current stresses | Burger King/Dairy Queen/local fast food job |
| Home/homeless - type of dwelling, number of stories, owned or rented | Lives at home with father and mother and one dog, Ruby |
| Financial situation- any current stresses | None. Stable home. |
| Insurance Status (e.g., un/under/insured, public/private, HMO/PPO) | Insured |
| Habits (i.e., diet, exercise, caffeine, smoking, alcohol, drugs) | Smoked marijuana once at a party over the last week |
| Activities (i.e., hobbies, sports, clubs, friends) | Very active, runs cross country |
| Typical day - what is the usual daily routine | AM practice, goes to school (has a car) classes until 315, cross country practice after school then home. Studies until around 10pm then goes to bed. Has data someone but they broke up 2 months ago. |

| CASE INFORMATION | |
| --- | --- |
| Chief Concern: What the patient will say when greeted by the student. The patient’s primary reason for seeking medical care often stated in his/own words. | Patient is there to get a refill on ADHD medication, but has learned that she will need to undergo drug screening. She does not want to do this because she recently tried marijuana. |
| Additional Concerns: Other, if any, concerns the patient has today (i.e., symptoms, requests, expectations, etc.) that will become part of set agenda. | The patient’s dad was planning to pick up the prescription later in the day, and if she doesn’t get the prescription, he will wonder why. |
|  | |
| THE PATIENT STORY: The SP will be asked to tell their symptom story and the personal and emotion impact for each of their concerns. You will want to write this is the patient voice. The symptom story should be able to answer this question: “Tell me more about [chief concern/additional concern], starting at the beginning and bringing me up to now.”  The personal context should be able to answer questions concerning the broader personal/psychosocial context of symptoms, especially the patient beliefs/attributions.  The emotional context should be able to ask how are you doing with this, how does this make you feel, how has this affected you emotionally? IMPACT: How has this affected your life? How has this been for your family? | I didn’t know there was a new policy. I do not want to give a urine sample today. Is there another way to get the prescription?  [You are evasive about why you will not comply with the request for the drug testing, but eventually reveal the reason.]  I tried marijuana 2 nights ago. I didn’t like it and promise to never do it again. I know you’ve made mistakes as a kid, can you cut me some slack and give me the prescription today? I know I’m going to test positive and then I’ll never get into medical school.  [If the resident does not want to give you a prescription, say:] But my dad is planning on picking up the prescription at the pharmacy today. If I don’t pass the test, he will wonder why the prescription isn’t there. I worry about what I will say to him.  [If the resident is insistent on you taking the drug test, say:] “Why would I give a urine sample? There is nothing in it for me. You can’t make me do this.” |
| HISTORY OF PRESENT ILLNESS: Although some of the HPI will be given in the patient’s symptom story, the learners will expand the story during the direct question section. Below describe the detailed history, usually about the chief concern, which the student must develop in order to make a useful assessment of the problem: | |
|  | |
| Onset (when; gradual or sudden) | Not applicable |
| Setting (what was going on or where was patient when symptoms first noticed?) | Not applicable |
| Duration (how long) | Not applicable |
| Time relationships (frequency, constant or intermittent) | Not applicable |
| Location | Not applicable |
| Radiation | Not applicable |
| Quality | Not applicable |
| Amount | Not applicable |
| Aggravated by what | Not applicable |
| Relieved by what | Not applicable |
| Associated with what | Not applicable |
| Attitude (what does the patient think is the problem, and how does he/she feel about it) | She did not know about the new drug screening policy, so she feels nervous about needing the prescription and not wanting to reveal that she tried marijuana. |
| Overall course | Not applicable |
| REVIEW OF SYSTEMS: Significant positives and negatives | |
|  |  |
|  |  |
|  |  |
|  |  |
|  | |
| Past medical history |  |
| Medication allergies (Name and reaction) | None |
| Environmental allergies (Name and reaction) | Hay Fever |
| Illnesses | No severe illnesses, cold/URIs |
| Vaccinations | UTD |
| Surgeries | None |
| Accidents/ injuries/ trauma | None |
| Hospitalization | Never |
|  | |
| Inclusive sexual and reproductive history | |
| Sexual practices  Sexual partners  Protection: Use of safer sex practices  Use of birth control if appropriate  Risk of intimate partner violence | Not sexually active. Has never had sex. Does know about contraception and birth control. If male, knows about condoms. |
| Ob/GYN HISTORY | Age of onset of menses 12 years old  Age of menopause Not applicable  Number of pregnancies None  Number of live births None  Number of miscarriages Not applicable  Number of abortions Not applicable |
| Medications | Prescription/dose/reason ADHD medication  Over the counter/dose/reason Tylenol for headaches  Herbs/supplements/dose/reason None  Other: |
| Immunizations | X Tetanus  X Flu  X Hepatitis  X Pneumovax  X HPV   - Other |
| Tobacco products:   - Cigarettes - Cigar - Pipe - Chew - E-cigarettes | X Never   - Past- year started/year quit - Current   - Quantity   - # of years |
| Alcohol   - Beer - Wine - Liquor - Other | X Never   - Past- year started/year quit - Current   - Quantity   - # of years |
| Drugs  X Weed   - Cocaine - Heroin - Meth - Other - IV - Inhalants - Other | - Never - Past- year started/year quit  1. Current    - Quantity  - # of years |
| Diet (describe) | Normal diet. No restrictions or limitations. |
| Exercise (describe) | Active, runs cross country |
| List any other important social history or information important to this case | Not applicable |
| Family history |  |
| Mother, Father, Siblings, Grandparents, and other significant findings. | Non-contributory |
|  |  |
| Physical Exam- List exam maneuvers expected for this case and any abnormal findings that SP will simulate. (tenderness, hyper-hypo reflex, rebound, weakness etc. )  Not applicable as this is a counseling case | |
| PHYSICAL EXAM FINDINGS |  |
| 1. Written in layman’s terms | Not applicable |
| 1. General appearance- affect, appearance, position of patient at opening (i.e. sitting, laying down, holding abdomen etc.) | Pleasant but nervous-appearing. Well-Developed/Well-nourished |
| 1. Vital signs | Appropriate for age |
| 1. Specific findings and affect | Not applicable |
| 1. Response to certain physical movements | Not applicable |
|  |  |
| DIAGNOSIS AND DIFFERENTIAL |  |
| Diagnosis with support from positive and negative history and PE findings | Not applicable |
| Differential with support from positive and negative history and PE findings | Not applicable |
|  |  |
| MANAGEMENT OR DIAGNOSTIC PLAN | There is no diagnosis required. The management plan is dependent on residents’ professionalism and ethical decision making in regards to next steps. |
|  |  |
| PROFESSIONALISM ISSUES OR CHALLENGES: | During the simulation, the resident will be challenged with balancing their sense of duty to the patient with their sense of the duty to the profession, which will be evidenced through reference to the new drug screening policy and standards of practice. |
