# Supplementary material for: Professionalism and Ethics: A Standardized Patient Observed Standardized Clinical Examination to Assess ACGME Pediatric Professionalism Milestones
Source: MedEdPORTAL. 2020 Jan 31;16:10873. doi: 10.15766/mep_2374-8265.10873 (PMC7062544; doi:10.15766/mep_2374-8265.10873)
Supplement: Supplementary file 1 — A. SP Case Development Tool Drug Screening.docx B. SP Case Development Tool Asthma.docx C. SP Case Development Tool Transfusion.docx D. SP Case Development Tool Mitochondrial.docx E. Door Notes.docx F. Learner Assessment Sheets.docx G. Debriefing Talking Points.docx H. Logistical Grid.docx I. Scenario Evaluations.docx J. OSCE Evaluation.docx K. Preevaluation for Preceptors.docx L. Postevaluation for Preceptors.docx [file mep-16-10873-s001.zip › B. SP Case Development Tool Asthma.docx]

Appendix B: *MedEdPORTAL* Standardized Patient Case Development Tool

Date: 5/9/19

Primary Case Author: Margaret Waltz, Arlene Davis, R. Jean Cadigan

Secondary Case Author: Rohit Jaswaney, Melissa Smith, Benny Joyner

Standardized Patient Educator: Melissa Smith and Benny Joyner

Name of Case: Asthma in the ED

Name of educational and or assessment activity: Professionalism and Ethics Simulation

Patient Name: William

Chief Complaint: Parent has a child with asthma exacerbation

Most likely Diagnosis and Differential with rationale from history and/or physical exam: The scenario is not about getting the correct diagnosis, but the decision process and the conversation with the parent.

Challenge question: Respond to the patient’s concerns about the drug screening and make a plan with her for next steps.

Domains: Check all that apply

X Professionalism

X Communication and Interpersonal skills

- Medical History
- Physical exam

X Shared Decision Making

- Patient Education
- Clinical Reasoning
- Documentation
- Handoff
- Presentation
- Other:

Type and level of learner: Pediatric residents at any level of training

Case Objectives: please list specific objectives for each of the domains you have checked above:

1. Identify at least one ethical issue related to professionalism in each case simulation

2. Apply ethical reasoning to arrive at an ethically permissible course of action

| SETTING: outpatient, in patient, ED, home, nursing home, rehab, group etc. | ED |
| --- | --- |
| PATIENT PROFILE: Information about the “patient” that helps select an SP and helps the learner get an understanding of them as a person. SP will know more information about the patient than learner will ever ask but allows SP to portray a fully developed patient personality. If none of the items below are particulars for the case please write “all may be used.” | |
| Age range | 30-55 years of age |
| Religious/spiritual background | Christian/Catholic |
| Sex (e.g., male, female, intersex, transwoman, transman) | Male |
| Sexual Orientation (e.g., heterosexual, lesbian, gay, bisexual, pansexual, queer, asexual) | Heterosexual |
| Gender expression (e.g., man, woman, gender queer) | Man |
| Race/ethnicity: | Any |
| Physical description (e.g., BMI, height range) | Any |
| Physical limitations | None |
| Patient appearance (e.g., disheveled, hospital gown, business casual, casual) | Casual |
| Moulage + location (e.g., none, bruises, scars, body piercing, tattoos) | None |
| Affect (e.g., pleasant, cooperative) | Frustrated; Strident |
| Family group (e.g., who is family, who they live with) | Wife at home, one other daughter. Has a son in the ED for asthma |
| Education | High school degree. |
| Level of health literacy | Low except for understanding that son needs albuterol when sick. Thinks that nebulizer “works better” than inhaler. Also, easier due to the fact that you can simply set it. |
| Employment, if any - present and past, noting any current stresses | Works as mechanic |
| Home/homeless - type of dwelling, number of stories, owned or rented | Lives in single family home that he rents. |
| Financial situation- any current stresses | Income around $45,000/year. Wife takes odd jobs babysitting. Total income approximately $55,000/year for family of four. Slightly stressed but has Medicaid. Financial stressors NOT contributing to issues. |
| Insurance Status (e.g., un/under/insured, public/private, HMO/PPO) | Medicaid |
| Habits (i.e., diet, exercise, caffeine, smoking, alcohol, drugs) | Smokes 1ppd. Occasional beer with friends/dinner. |
| Activities (i.e., hobbies, sports, clubs, friends) | Father participates in weekend soccer. Child is moderately active in after school activities but nothing organized due to asthma. |
| Typical day - what is the usual daily routine | Father works M-F 7am-5pm. Son in 4^th^ grade. Daughter stays at home with mother (daughter is 4 years old). |

| CASE INFORMATION | |
| --- | --- |
| Chief Concern: What the patient will say when greeted by the student. The patient’s primary reason for seeking medical care often stated in his/own words. | The parent of the 10 year-old patient says that he needs a prescription for a nebulizer. They lost his son’s nebulizer in a recent move. |
| Additional Concerns: Other, if any, concerns the patient has today (i.e., symptoms, requests, expectations, etc.) that will become part of set agenda. | The patient tried before to use an inhaler, but it didn’t work. The patient ended up in the ICU. |
|  | |
| THE PATIENT STORY: The SP will be asked to tell their symptom story and the personal and emotion impact for each of their concerns. You will want to write this is the patient voice. The symptom story should be able to answer this question: “Tell me more about [chief concern/additional concern], starting at the beginning and bringing me up to now.”  The personal context should be able to answer questions concerning the broader personal/psychosocial context of symptoms, especially the patient beliefs/attributions.  The emotional context should be able to ask how are you doing with this, how does this make you feel, how has this affected you emotionally? IMPACT: How has this affected your life? How has this been for your family? | My son’s asthma is acting up. We’ve just moved and we lost the nebulizer in the move. I need him to have a nebulizer treatment. I don’t want an inhaler for him. The inhaler doesn’t work. The last time we used the inhaler, my son ended up in the ICU. The only thing that works is the nebulizer. |
| HISTORY OF PRESENT ILLNESS: Although some of the HPI will be given in the patient’s symptom story, the learners will expand the story during the direct question section. Below describe the detailed history, usually about the chief concern, which the student must develop in order to make a useful assessment of the problem: | |
|  | |
| Onset (when; gradual or sudden) | 4 hours prior to admission to ED |
| Setting (what was going on or where was patient when symptoms first noticed?) | At home, getting ready for bed |
| Duration (how long) | 4 hours |
| Time relationships (frequency, constant or intermittent) | Constant wheeze |
| Location | Bilateral lung fields |
| Radiation | Throughout all lung fields |
| Quality |  |
| Amount |  |
| Aggravated by what | Cold, uri |
| Relieved by what | albuterol |
| Associated with what |  |
| Attitude (what does the patient think is the problem, and how does he/she feel about it) |  |
| Overall course |  |
| REVIEW OF SYSTEMS: Significant positives and negatives | |
|  | +cough, runny nose for past 1-2 days, no gi symptoms, no fever |
|  |  |
|  |  |
|  |  |
|  | |
| Past medical history **(for patient not parent)** |  |
| Medication allergies (Name and reaction) | None |
| Environmental allergies (Name and reaction) | Hay fever (allergies to pet dander, some grasses/trees) |
| Illnesses | Asthma attack (status asthmaticus) ~ 1-2 times/year |
| Vaccinations | Up to date (did get flu vaccine) |
| Surgeries | None |
| Accidents/ injuries/ trauma | None |
| Hospitalization | Prior hospitalization in the PICU for asthma-related complications |
|  | |
| Inclusive sexual and reproductive history | |
| Sexual practices  Sexual partners  Protection: Use of safer sex practices  Use of birth control if appropriate  Risk of intimate partner violence | Not applicable |
| Ob/GYN HISTORY | Age of onset of menses Not applicable  Age of menopause Not applicable  Number of pregnancies Not applicable  Number of live births Not applicable  Number of miscarriages Not applicable  Number of abortions Not applicable |
| Medications | Prescription/dose/reason: albuterol nebulizer  Over the counter/dose/reason: typlenol prn fever  Herbs/supplements/dose/reason  Other: |
| Immunizations (Up to date) | - Tetanus - Flu - Hepatitis - Pneumovax - HPV   X Other (UTD) |
| Tobacco products:   - Cigarettes - Cigar - Pipe - Chew - E-cigarettes | xNever   - Past- year started/year quit - Current   - Quantity   - # of years |
| Alcohol   - Beer - Wine - Liquor - Other | xNever   - Past- year started/year quit - Current   - Quantity   - # of years |
| Drugs   - Weed - Cocaine - Heroin - Meth - Other - IV - Inhalants - Other | xNever   - Past- year started/year quit - Current   - Quantity - # of years |
| Diet (describe) | Normal |
| Exercise (describe) | Active child. No active sports |
| List any other important social history or information important to this case | Recent move to current location (~6 months ago). Working on establishing ties. Father just started new job. Asthma exacerbations for child have increased-father frustrated. Just wants nebulizer because “that’s what we used before.” |
| Family history |  |
| Mother, Father, Siblings, Grandparents, and other significant findings. | Non contributory |
|  |  |
| Physical Exam- List exam maneuvers expected for this case and any abnormal findings that SP will simulate. (tenderness, hyper-hypo reflex, rebound, weakness etc. )  Child with wheezes and shortness of breath; no child to be examined. | |
| PHYSICAL EXAM FINDINGS |  |
| 1. Written in layman’s terms | Not applicable |
| 1. General appearance- affect, appearance, position of patient at opening (i.e. sitting, laying down, holding abdomen etc.) | Child with anxious appearing affect, however, no physicial child to be examined. |
| 1. Vital signs | Not applicable |
| 1. Specific findings and affect | Not applicable |
| 1. Response to certain physical movements | Not applicable |
|  |  |
| DIAGNOSIS AND DIFFERENTIAL |  |
| Diagnosis with support from positive and negative history and PE findings | Not applicable |
| Differential with support from positive and negative history and PE findings | Not applicable |
|  |  |
| MANAGEMENT OR DIAGNOSITIC PLAN | Child with asthma. The management plan is dependent on residents’ professionalism and ethical decision making in regards to next steps (nebulizer vs metered dose inhaler). |
|  |  |
| PROFESSIONALISM ISSUES OR CHALLENGES: | In this simulation, the resident will be challenged with displaying empathy and compassion and an understanding of patient and family experiences. And work toward negotiating a solution with the family about albuterol delivery device. |
