# Supplementary material for: Professionalism and Ethics: A Standardized Patient Observed Standardized Clinical Examination to Assess ACGME Pediatric Professionalism Milestones
Source: MedEdPORTAL. 2020 Jan 31;16:10873. doi: 10.15766/mep_2374-8265.10873 (PMC7062544; doi:10.15766/mep_2374-8265.10873)
Supplement: Supplementary file 1 — A. SP Case Development Tool Drug Screening.docx B. SP Case Development Tool Asthma.docx C. SP Case Development Tool Transfusion.docx D. SP Case Development Tool Mitochondrial.docx E. Door Notes.docx F. Learner Assessment Sheets.docx G. Debriefing Talking Points.docx H. Logistical Grid.docx I. Scenario Evaluations.docx J. OSCE Evaluation.docx K. Preevaluation for Preceptors.docx L. Postevaluation for Preceptors.docx [file mep-16-10873-s001.zip › C. SP Case Development Tool Transfusion.docx]

Appendix C: *MedEdPORTAL* Standardized Patient Case Development Tool

Date: 5/9/19

Primary Case Author: Margaret Waltz, Arlene Davis, R. Jean Cadigan

Secondary Case Author: Rohit Jaswaney, Melissa Smith, Benny Joyner

Standardized Patient Educator: Melissa Smith and Benny Joyner

Name of Case: Transfusion in the Hospital

Name of educational and or assessment activity: Professionalism and Ethics Simulation

Patient Name: Rosemary

Chief Complaint: Parent has a child with pancytopenia

Most likely Diagnosis and Differential with rationale from history and/or physical exam: The scenario is not about getting the correct diagnosis, but the decision process and the conversation with the parent.

Challenge question: Respond to the parent’s concern and develop a plan for the next steps to take, given her son’s condition.

Domains: Check all that apply

X Professionalism

X Communication and Interpersonal skills

- Medical History
- Physical exam

X Shared Decision Making

- Patient Education
- Clinical Reasoning
- Documentation
- Handoff
- Presentation
- Other:

Type and level of learner: Pediatric residents at any level of training

Case Objectives: please list specific objectives for each of the domains you have checked above:

1. Identify at least one ethical issue related to professionalism in each case simulation

2. Apply ethical reasoning to arrive at an ethically permissible course of action

| SETTING: outpatient, in patient, ED, home, nursing home, rehab, group etc. | In patient |
| --- | --- |
| PATIENT PROFILE: Information about the “patient” that helps select an SP and helps the learner get an understanding of them as a person. SP will know more information about the patient than learner will ever ask but allows SP to portray a fully developed patient personality. If none of the items below are particulars for the case please write “all may be used.” | |
| Age range | 35-45 years of age |
| Religious/spiritual background | Jehovah’s Witness |
| Sex (e.g., male, female, intersex, transwoman, transman) | All may be used, and a suitable name may be chosen |
| Sexual Orientation (e.g., heterosexual, lesbian, gay, bisexual, pansexual, queer, asexual) | Heterosexual |
| Gender expression (e.g., man, woman, gender queer) | Female |
| Race/ethnicity: | African American preferred but any are suitable |
| Physical description (e.g., BMI, height range) | Any |
| Physical limitations | None |
| Patient appearance (e.g., disheveled, hospital gown, business casual, casual) | Casual |
| Moulage + location (e.g., none, bruises, scars, body piercing, tattoos) | None |
| Affect (e.g., pleasant, cooperative) | Worried and frustrated |
| Family group (e.g., who is family, who they live with) | Has a son who has spent the night in the hospital, needing a transfusion |
| Education | Some college |
| Level of health literacy | Moderate. Able to understand basics of health care but no sophistication regarding medicine. |
| Employment, if any - present and past, noting any current stresses | Works as a cashier at the local grocery store |
| Home/homeless - type of dwelling, number of stories, owned or rented | Rents apartment |
| Financial situation- any current stresses | Lives paycheck to paycheck. No appreciable savings generated but finances are not a stressor. “God will provide” |
| Insurance Status (e.g., un/under/insured, public/private, HMO/PPO) | Commercial insurance |
| Habits (i.e., diet, exercise, caffeine, smoking, alcohol, drugs) | Normal diet. Minimal exercise. Does not smoke or drink. |
| Activities (i.e., hobbies, sports, clubs, friends) | Church is her primary social outlet. |
| Typical day - what is the usual daily routine | Up to get son ready for school. Prayer in the morning before going to work. Works until approximately 4pm. Comes home, prepares dinner. Reads bible before going to bed. |

| CASE INFORMATION | |
| --- | --- |
| Chief Concern: What the patient will say when greeted by the student. The patient’s primary reason for seeking medical care often stated in his/own words. | The parent was told her son needs a transfusion. Because of her Jehovah’s Witness faith, she does not want him to have any blood products. She is frustrated after the team threatened her with a court order to give her son the transfusion. |
| Additional Concerns: Other, if any, concerns the patient has today (i.e., symptoms, requests, expectations, etc.) that will become part of set agenda. | Not applicable |
|  | |
| THE PATIENT STORY: The SP will be asked to tell their symptom story and the personal and emotion impact for each of their concerns. You will want to write this is the patient voice. The symptom story should be able to answer this question: “Tell me more about [chief concern/additional concern], starting at the beginning and bringing me up to now.”  The personal context should be able to answer questions concerning the broader personal/psychosocial context of symptoms, especially the patient beliefs/attributions.  The emotional context should be able to ask how are you doing with this, how does this make you feel, how has this affected you emotionally? IMPACT: How has this affected your life? How has this been for your family? | I don’t want him to have a transfusion. Our faith won’t allow that. You all are trying to take our rights away. He may come around!  [As the resident explains why the transfusion is necessary, say:]  Why do we have to do that now? Why can’t we wait? I need more time. This is a big decision to have to make. Is there any possibility he could get better without a transfusion? Can we wait for that.  [If the resident is understanding, say:]  I won’t stop you from giving him a transfusion if it is absolutely necessary, but I won’t give you my permission. |
| HISTORY OF PRESENT ILLNESS: Although some of the HPI will be given in the patient’s symptom story, the learners will expand the story during the direct question section. Below describe the detailed history, usually about the chief concern, which the student must develop in order to make a useful assessment of the problem: | |
|  | |
| Onset (when; gradual or sudden) | gradual |
| Setting (what was going on or where was patient when symptoms first noticed?) | Pancytopenia in the setting of myelodysplastic syndrome |
| Duration (how long) | Over 12 hours |
| Time relationships (frequency, constant or intermittent) |  |
| Location |  |
| Radiation |  |
| Quality |  |
| Amount | 10 g/dL (hct 30%)🡪 7.5 g/dL (hct 23%) |
| Aggravated by what |  |
| Relieved by what |  |
| Associated with what |  |
| Attitude (what does the patient think is the problem, and how does he/she feel about it) |  |
| Overall course | Worsening mental status (awake but somnolent) |
| REVIEW OF SYSTEMS: Significant positives and negatives | |
|  |  |
|  |  |
|  |  |
|  |  |
|  | |
| Past medical history |  |
| Medication allergies (Name and reaction) | None |
| Environmental allergies (Name and reaction) | None |
| Illnesses | None |
| Vaccinations | Up to date |
| Surgeries | None |
| Accidents/ injuries/ trauma | None |
| Hospitalization | None |
|  | |
| Inclusive sexual and reproductive history | |
| Sexual practices  Sexual partners  Protection: Use of safer sex practices  Use of birth control if appropriate  Risk of intimate partner violence | Not applicable |
| Ob/GYN HISTORY | Age of onset of menses Not applicable  Age of menopause Not applicable  Number of pregnancies Not applicable  Number of live births Not applicable  Number of miscarriages Not applicable  Number of abortions Not applicable |
| Medications | Prescription/dose/reason Not applicable  Over the counter/dose/reason Not applicable  Herbs/supplements/dose/reason Not applicable  Other: |
| Immunizations (Up to date) | - Tetanus - Flu - Hepatitis - Pneumovax - HPV - Other |
| Tobacco products:   - Cigarettes - Cigar - Pipe - Chew - E-cigarettes | X Never   - Past- year started/year quit - Current   - Quantity   - # of years |
| Alcohol   - Beer - Wine - Liquor - Other | X Never   - Past- year started/year quit - Current   - Quantity   - # of years |
| Drugs   - Weed - Cocaine - Heroin - Meth - Other - IV - Inhalants - Other | X Never   - Past- year started/year quit - Current   - Quantity - # of years |
| Diet (describe) | Not applicable |
| Exercise (describe) | Not applicable |
| List any other important social history or information important to this case | Not applicable |
| Family history |  |
| Mother, Father, Siblings, Grandparents, and other significant findings. | Not applicable |
|  |  |
| Physical Exam- List exam maneuvers expected for this case and any abnormal findings that SP will simulate. (tenderness, hyper-hypo reflex, rebound, weakness etc. )  Not applicable | |
| PHYSICAL EXAM FINDINGS |  |
| 1. Written in layman’s terms | Not applicable |
| 1. General appearance- affect, appearance, position of patient at opening (i.e. sitting, laying down, holding abdomen etc.) | Not applicable |
| 1. Vital signs | Not applicable |
| 1. Specific findings and affect | Not applicable |
| 1. Response to certain physical movements | Not applicable |
|  |  |
| DIAGNOSIS AND DIFFERENTIAL |  |
| Diagnosis with support from positive and negative history and PE findings | Not applicable |
| Differential with support from positive and negative history and PE findings | Not applicable |
|  |  |
| MANAGEMENT OR DIAGNOSITIC PLAN | There is no diagnosis required. The management plan is dependent on residents’ professionalism and ethical decision making in regards to next steps. |
|  |  |
| PROFESSIONALISM ISSUES OR CHALLENGES: | In this simulation, the resident will be challenged with aligning therapeutic care with the parent’s goals and values in the context of labs that are uncertain in terms of urgency of action. |
