# Supplementary material for: Professionalism and Ethics: A Standardized Patient Observed Standardized Clinical Examination to Assess ACGME Pediatric Professionalism Milestones
Source: MedEdPORTAL. 2020 Jan 31;16:10873. doi: 10.15766/mep_2374-8265.10873 (PMC7062544; doi:10.15766/mep_2374-8265.10873)
Supplement: Supplementary file 1 — A. SP Case Development Tool Drug Screening.docx B. SP Case Development Tool Asthma.docx C. SP Case Development Tool Transfusion.docx D. SP Case Development Tool Mitochondrial.docx E. Door Notes.docx F. Learner Assessment Sheets.docx G. Debriefing Talking Points.docx H. Logistical Grid.docx I. Scenario Evaluations.docx J. OSCE Evaluation.docx K. Preevaluation for Preceptors.docx L. Postevaluation for Preceptors.docx [file mep-16-10873-s001.zip › D. SP Case Development Tool Mitochondrial.docx]

Appendix D: *MedEdPORTAL* Standardized Patient Case Development Tool

Date: 5/9/19

Primary Case Author: Margaret Waltz, Arlene Davis, R. Jean Cadigan

Secondary Case Author: Rohit Jaswaney, Melissa Smith, Benny Joyner

Standardized Patient Educator: Melissa Smith and Benny Joyner

Name of Case: Mitochondrial Disorder

Name of educational and or assessment activity: Professionalism and Ethics Simulation

Patient Name: Marie

Chief Complaint: Parent has a child with mitochondrial disorder who hasn’t received her medication during her hospital stay

Most likely Diagnosis and Differential with rationale from history and/or physical exam: The scenario is not about getting the correct diagnosis, but the decision process and the conversation with the parent.

Challenge question: Respond to the mother’s concern about the patient and make a plan with her for next steps.

Domains: Check all that apply

X Professionalism

X Communication and Interpersonal skills

- Medical History
- Physical exam

X Shared Decision Making

- Patient Education
- Clinical Reasoning
- Documentation
- Handoff
- Presentation
- Other:

Type and level of learner: Pediatric residents at any level of training

Case Objectives: please list specific objectives for each of the domains you have checked above:

1. Identify at least one ethical issue related to professionalism in each case simulation

2. Apply ethical reasoning to arrive at an ethically permissible course of action

| SETTING: outpatient, in patient, ED, home, nursing home, rehab, group etc. | In patient |
| --- | --- |
| PATIENT PROFILE: Information about the “patient” that helps select an SP and helps the learner get an understanding of them as a person. SP will know more information about the patient than learner will ever ask but allows SP to portray a fully developed patient personality. If none of the items below are particulars for the case please write “all may be used.” | |
| Age range | 25-45 years old |
| Religious/spiritual background | Christian |
| Sex (e.g., male, female, intersex, transwoman, transman) | All may be used, and a suitable name may be chosen |
| Sexual Orientation (e.g., heterosexual, lesbian, gay, bisexual, pansexual, queer, asexual) | Heterosexual |
| Gender expression (e.g., man, woman, gender queer) | All may be used |
| Race/ethnicity: | All may be used |
| Physical description (e.g., BMI, height range) | All may be used |
| Physical limitations | None |
| Patient appearance (e.g., disheveled, hospital gown, business casual, casual) | Casual, tired appearing |
| Moulage + location (e.g., none, bruises, scars, body piercing, tattoos) | Not applicable |
| Affect (e.g., pleasant, cooperative) | Worried, frustrated, upset |
| Family group (e.g., who is family, who they live with) | Has a daughter with a mitochondrial disorder in the hospital |
| Education | College graduate |
| Level of health literacy | High level of literacy, very familiar with mitochondrial disorder and their unusual dietary requirements |
| Employment, if any - present and past, noting any current stresses | Stay at home mom. Was a certified nurse assistant until child was born. Now stays at home caring for child. |
| Home/homeless - type of dwelling, number of stories, owned or rented | Single family dwelling. 2 bedrooms, 2 bathroom single level home in a rural location. |
| Financial situation- any current stresses | Husband works in sales at local office supply company. |
| Insurance Status (e.g., un/under/insured, public/private, HMO/PPO) | Commercial insurance. |
| Habits (i.e., diet, exercise, caffeine, smoking, alcohol, drugs) | Normal diet. Minimal exercise. |
| Activities (i.e., hobbies, sports, clubs, friends) | Limited circle of friends-primarily consists of women at the local diner that she knows through church |
| Typical day - what is the usual daily routine | Daily routine revolves around caring for her daughter. Up by 630 AM and usually awake until late evening 10/1030pm. Has home health nurse overnight but the service is not reliable. |

| CASE INFORMATION | |
| --- | --- |
| Chief Concern: What the patient will say when greeted by the student. The patient’s primary reason for seeking medical care often stated in his/own words. | Parent is upset that the daughter has not received her medication, which the parent brought from home in an unmarked bottle. It is against hospital policy to give unlabeled medications, so the daughter has missed a dose. The parent is worry this will cause further neurologic damage. |
| Additional Concerns: Other, if any, concerns the patient has today (i.e., symptoms, requests, expectations, etc.) that will become part of set agenda. |  |
|  | |
| THE PATIENT STORY: The SP will be asked to tell their symptom story and the personal and emotion impact for each of their concerns. You will want to write this is the patient voice. The symptom story should be able to answer this question: “Tell me more about [chief concern/additional concern], starting at the beginning and bringing me up to now.”  The personal context should be able to answer questions concerning the broader personal/psychosocial context of symptoms, especially the patient beliefs/attributions.  The emotional context should be able to ask how are you doing with this, how does this make you feel, how has this affected you emotionally? IMPACT: How has this affected your life? How has this been for your family? | My daughter has missed a dose of her medication! We can’t change her medication schedule. It could cause her to have neurological damage. The nutritionist I spoke to assured me the medication would be given to her. Why hasn’t anyone given her the dose?  [As resident explains, say:]  What will happen to my daughter if we miss another dose? She is already less energetic and less like herself.  [If resident is insistent that they won’t give the medication, say:]  I don’t care about your policy. I’m not comfortable with that plan. What else are we going to do?  [At the end of the conversation, ask:]  How are you going to make sure this doesn’t happen to my daughter in the future? |
| HISTORY OF PRESENT ILLNESS: Although some of the HPI will be given in the patient’s symptom story, the learners will expand the story during the direct question section. Below describe the detailed history, usually about the chief concern, which the student must develop in order to make a useful assessment of the problem: | |
|  | |
| Onset (when; gradual or sudden) | gradual |
| Setting (what was going on or where was patient when symptoms first noticed?) | At home with fever and diarrhea |
| Duration (how long) | 12 hours prior to admission |
| Time relationships (frequency, constant or intermittent) |  |
| Location |  |
| Radiation |  |
| Quality |  |
| Amount |  |
| Aggravated by what |  |
| Relieved by what |  |
| Associated with what |  |
| Attitude (what does the patient think is the problem, and how does he/she feel about it) |  |
| Overall course |  |
| REVIEW OF SYSTEMS: Significant positives and negatives | |
|  |  |
|  |  |
|  |  |
|  |  |
|  | |
| Past medical history |  |
| Medication allergies (Name and reaction) | None |
| Environmental allergies (Name and reaction) | None |
| Illnesses | Frequent colds and has ended up hospitalized on multiple occasions secondary to mitochondrial disorder. Patient and mother known to the service and to the genetic/metabolic team. |
| Vaccinations | Up to Date |
| Surgeries | Gastrostomy tube placement 4 years ago |
| Accidents/ injuries/ trauma | Not applicable |
| Hospitalization | Multiple hospitalizations. Most recently 3 months prior to this visit. |
|  | |
| Inclusive sexual and reproductive history | |
| Sexual practices  Sexual partners  Protection: Use of safer sex practices  Use of birth control if appropriate  Risk of intimate partner violence | Not applicable |
| Ob/GYN HISTORY | Age of onset of menses Not applicable  Age of menopause Not applicable  Number of pregnancies Not applicable  Number of live births Not applicable  Number of miscarriages Not applicable  Number of abortions Not applicable |
| Medications | Prescription/dose/reason Daughter takes Ubiquinone 100 mg q12  Over the counter/dose/reason  Herbs/supplements/dose/reason  Other: |
| Immunizations | - Tetanus - Flu - Hepatitis - Pneumovax - HPV - Other |
| Tobacco products:   - Cigarettes - Cigar - Pipe - Chew - E-cigarettes | X Never   - Past- year started/year quit - Current   - Quantity   - # of years |
| Alcohol   - Beer - Wine - Liquor - Other | X Never   - Past- year started/year quit - Current   - Quantity   - # of years |
| Drugs   - Weed - Cocaine - Heroin - Meth - Other - IV - Inhalants - Other | X Never   - Past- year started/year quit - Current   - Quantity - # of years |
| Diet (describe) | High protein diet |
| Exercise (describe) | None |
| List any other important social history or information important to this case | Child lives with mother and father at home. This child is the mother’s entire world. |
| Family history |  |
| Mother, Father, Siblings, Grandparents, and other significant findings. | Non-contributory |
|  |  |
| Physical Exam- List exam maneuvers expected for this case and any abnormal findings that SP will simulate. (tenderness, hyper-hypo reflex, rebound, weakness etc. )  Not applicable | |
| PHYSICAL EXAM FINDINGS |  |
| 1. Written in layman’s terms | Not applicable |
| 1. General appearance- affect, appearance, position of patient at opening (i.e. sitting, laying down, holding abdomen etc.) | Anxious appearing and upset mother |
| 1. Vital signs | Not applicable |
| 1. Specific findings and affect | Not applicable |
| 1. Response to certain physical movements | Not applicable |
|  |  |
| DIAGNOSIS AND DIFFERENTIAL |  |
| Diagnosis with support from positive and negative history and PE findings | Not applicable |
| Differential with support from positive and negative history and PE findings | Not applicable |
|  |  |
| MANAGEMENT OR DIAGNOSITIC PLAN | There is no diagnosis required. The management plan is dependent on residents’ professionalism and ethical decision making in regards to next steps. |
|  |  |
| PROFESSIONALISM ISSUES OR CHALLENGES: | In this simulation, the resident will be challenged with communicating empathetically with the parent while conveying insight into the team’s actions and hospital policy. |
