# Supplementary material for: Professionalism and Ethics: A Standardized Patient Observed Standardized Clinical Examination to Assess ACGME Pediatric Professionalism Milestones
Source: MedEdPORTAL. 2020 Jan 31;16:10873. doi: 10.15766/mep_2374-8265.10873 (PMC7062544; doi:10.15766/mep_2374-8265.10873)
Supplement: Supplementary file 1 — A. SP Case Development Tool Drug Screening.docx B. SP Case Development Tool Asthma.docx C. SP Case Development Tool Transfusion.docx D. SP Case Development Tool Mitochondrial.docx E. Door Notes.docx F. Learner Assessment Sheets.docx G. Debriefing Talking Points.docx H. Logistical Grid.docx I. Scenario Evaluations.docx J. OSCE Evaluation.docx K. Preevaluation for Preceptors.docx L. Postevaluation for Preceptors.docx [file mep-16-10873-s001.zip › E. Door Notes.docx]

**Drug Screening Case in the Clinic: Copy for Resident**

You are the resident being precepted in continuity clinic. Emily, a 17-year-old, is your next patient. She has ongoing issues with ADHD, asthma, and eczema. She is seen in the clinic frequently (on average every 2-3 months for the past 18 months related to her asthma and eczema), although this is her first time seeing you. Overall, she is an active kid, running cross country, and is an excellent student (As and Bs in school). She hopes to be a psychiatrist one day.

Emily has been on ADHD controller medication consistently for the last several years without significant issues. A new clinic policy requires drug testing for all patients with prescriptions for controlled substances, including Emily’s prescribed ADHD controller medication. Under the policy, patients should be drug tested upon arrival at every clinic visit. Patients with less than therapeutic levels or patients who test positive for non-prescription controlled substances or illicit substances will not receive a prescription that day. The policy is to ensure the drug is being used by the patient (not diverted) and that the patient isn’t using other substances that interfere with treatment.

This is Emily’s first visit since the policy went into place. You have been told that every patient in the clinic has been sent a copy of the policy. As you go in to see Emily, you notice that she has not provided the urine sample (chart simply says: did not obtain). Emily would like to speak to you about the drug testing and the prescription she needs.

You must now respond to your patient’s concerns and make a plan with her for next steps.

**Drug Screening Case in the Clinic: Copy for Patient Actor**

*Scenario for Resident Dr:*

You are the resident being precepted in continuity clinic. Emily, a 17-year-old, is your next patient. She has ongoing issues with ADHD, asthma, and eczema. She is seen in the clinic frequently (on average every 2-3 months for the past 18 months related to her asthma and eczema), although this is her first time seeing you. Overall, she is an active kid, running cross country, and is an excellent student (As and Bs in school). She hopes to be a psychiatrist one day.

Emily has been on ADHD controller medication consistently for the last several years without significant issues. A new clinic policy requires drug testing for all patients with prescriptions for controlled substances, including Emily’s prescribed ADHD controller medication. Under the policy, patients should be drug tested upon arrival at every clinic visit. Patients with less than therapeutic levels or patients who test positive for non-prescription controlled substances or illicit substances will not receive a prescription that day. The policy is to ensure the drug is being used by the patient (not diverted) and that the patient isn’t using other substances that interfere with treatment.

This is Emily’s first visit since the policy went into place. You have been told that every patient in the clinic has been sent a copy of the policy. As you go in to see Emily, you notice that she has not provided the urine sample (chart simply says: did not obtain). Emily would like to speak to you about the drug testing and the prescription she needs.

You must now respond to your patient’s concerns and make a plan with her for next steps.

*How to direct the conversation:*

When asked why you will not comply with the request for drug testing, you are evasive but eventually reveal that you tried marijuana 2 nights ago. You say you didn’t like it and promise to never do it again. You also say to the resident: “I know you’ve made mistakes as a kid, can you cut me some slack and give me the prescription today? I know I’m going to test positive and then I’ll never get into medical school.” Then if the resident does not want to give you a prescription, say “But my dad is planning on picking up the prescription at the pharmacy today. If I don’t pass the test, he will wonder why the prescription isn’t there.” You may add that you worry about what you will say to him. If the resident is insistent on you taking the drug test, you may say “Why would I give a urine sample? There is nothing in it for me. You can’t make me do this.”

*Resident’s objective*:

Resident must respond to your questions and make a plan for the next steps.

**Asthma Case in the ED: Copy for Resident**

You are a resident in the ED when a 10-year-old male with a history of mild intermittent asthma comes in at 1 am with a 4-hour history of shortness of breath with bilateral wheezes.  His O2 saturations are 95%, and RR 35. His parent tells you that he uses a nebulizer at home, but it was lost in a recent move. You order 8 puffs of an Albuterol 90 mcg MDI, but when the nurse attempts to administer the medication, the parent refuses the inhaler, requesting a nebulized albuterol treatment instead. The parent also wants to go home with a prescription for a nebulizer.

You must respond to the parent’s concerns and make a plan with the parent.

**Asthma Case in the ED: Copy for Patient Actor**

*Scenario for Resident Dr:*

You are a resident in the ED when a 10-year-old male with a history of mild intermittent asthma comes in at 1 am with a 4-hour history of shortness of breath with bilateral wheezes. His O2 saturations are 95%, and RR 35. His parent tells you that he uses a nebulizer at home, but it was lost in a recent move. You order 8 puffs of an Albuterol 90 mcg MDI, but when the nurse attempts to administer the medication, the parent refuses the inhaler, requesting a nebulized albuterol treatment instead. The parent also wants to go home with a prescription for a nebulizer.

You must respond to the parent’s concerns and make a plan with the parent.

*How to direct the conversation:*

As the parent of the patient, you are adamant that your son be given a nebulizer treatment rather than an inhaler (MDI). Share with the resident that the last time your son had an asthma flare, an inhaler was tried first, and then your son ended up being admitted to the PICU. You believe he didn’t improve until he had nebulized medications. You remain adamant about receiving a nebulized treatment and prescription for the nebulizer. If the resident is insistent on the puffer, you can say “I don’t think you are hearing me” while remaining adamant about the nebulizer. If resident adequately listens and responds to your questions and concerns, then you can agree to the inhaler in the ED. You can choose to negotiate with the resident about what the prescription for home will be (nebulizer or inhaler).

*Resident’s objective*:

Resident must respond to your concerns and make a plan for the next steps.

**Transfusion Case in the Hospital: Copy for Resident**

You are working days on HemOnc and your patients include a 14-year-old boy with pancytopenia after a recent diagnosis of myelodysplastic syndrome. The patient’s mother refuses blood products for him, stating that they are of the Jehovah’s Witness faith. The patient’s counts are dropping from 10 g/dL and HCT at 30% 12 hours ago, and hemoglobin is now 7.5 g/dL, HCT is 23%, and platelet count is 55. The patient is awake and conversant, but will drift off to sleep fairly quickly. The mother is very upset because the overnight physician threatened to get a court order for a transfusion. “You’re trying to take away my rights,” she has told the team in frustration. The team knows that a transfusion will soon be necessary because nonblood products will not be an appropriate alternative for the falling blood count, but the team keeps hoping the mother “will come around” and agree to it as she watches her son get weaker. She has told the team that she just wants her son to be ok, but she cannot permit a blood transfusion.

*You must talk with her about next steps, given her son’s condition.*

**Transfusion Case in the Hospital: Copy for Patient Actor**

*Scenario for Resident Dr:*

You are working days on HemOnc and your patients include a 14-year-old boy with pancytopenia after a recent diagnosis of myelodysplastic syndrome. The patient’s mother refuses blood products for him, stating that they are of the Jehovah’s Witness faith. The patient’s counts are dropping from 10 g/dL and HCT at 30% 12 hours ago, and hemoglobin is now 7.5 g/dL, HCT is 23%, and platelet count is 55. The patient is awake and conversant, but will drift off to sleep fairly quickly. The mother is very upset because the overnight physician threatened to get a court order for a transfusion. “You’re trying to take away my rights,” she has told the team in frustration. The team knows that a transfusion will soon be necessary because nonblood products will not be an appropriate alternative for the falling blood count, but the team keeps hoping the mother “will come around” and agree to it as she watches her son get weaker. She has told the team that she just wants her son to be ok, but she cannot permit a blood transfusion.

*How to direct the conversation:*

Resident will come in to talk to you about your son’s condition. You should push back about your belief system and that you do not want blood product transfusions. You should also push back and say “Why now? Why can’t we wait? I need more time. This is a big decision to have to make.” You may also ask, “Is there any possibility he could get better without a transfusion? Can we wait for that?” Eventually if the resident is understanding, you can agree that you will not stop the transfusion if it is absolutely necessary, but that the hospital will have to give it without your permission.

*Resident’s objective*:

Resident must respond to your questions and make a plan for the next steps.

**Mitochondrial Disorder Case: Copy for Resident**

You are the overnight resident for the general floor team and are taking care of a 2-year-old girl with a mitochondrial disorder who was admitted earlier that day for fever and diarrhea. At 9 pm her nurse calls to say that her mother is here and upset that her daughter has not received her home (non-formulary) medication of Ubiquinone (an oxidized form of Coenzyme Q-10). The mother states that the staff nutritionist had assured her that it would be given with her evening meal. The medication is a white powder in an unmarked bottle at the child’s bedside. It is against hospital policy to give medication that is unlabeled.

You must now respond to the mother’s concerns and make a plan for next steps.

**Mitochondrial Disorder Case: Copy for Patient Actor**

*Scenario for Resident Dr:*

You are the overnight resident for the general floor team and are taking care of a 2-year-old girl with a mitochondrial disorder who was admitted earlier that day for fever and diarrhea. At 9 pm her nurse calls to say that her mother is here and upset that her daughter has not received her home (non-formulary) medication of Ubiquinone (an oxidized form of Coenzyme Q-10). The mother states that the staff nutritionist had assured her that it would be given with her evening meal.  The medication is a white powder in an unmarked bottle at the child’s bedside. It is against hospital policy to give medication that is unlabeled.

You must now respond to the mother’s concerns and make a plan for next steps.

*How to direct the conversation:*

The resident comes in to talk with you. You state that you are upset that your daughter has missed the Ubiquinone medication because any change in her medication schedule can cause further neurologic damage in her mitochondrial disease process. You are adamant that you had already spoken to the genetic nutritionist earlier in the day who said the medication would be given. You are upset the day shift staff messed up and did not give her the dose with her dinner even though the nutritionist okayed it. During the discussion, you should ask the resident “Why hasn’t anyone given her the dose?” and “What will happen to my daughter if we miss another dose? She is already less energetic and less like herself.” If the resident is insistent and inflexible during the discussion in that the resident won’t give the medicine, you can push back by saying, “I don’t care about your policy. I’m not comfortable with that plan. What else are we going to do?” Regardless of pushback, you should ask the resident how to correct the problem and how to make sure your daughter can receive it in the future.

*Resident’s objective*:

Resident must respond to your questions and make a plan for the next steps.
