# Supplementary material for: Professionalism and Ethics: A Standardized Patient Observed Standardized Clinical Examination to Assess ACGME Pediatric Professionalism Milestones
Source: MedEdPORTAL. 2020 Jan 31;16:10873. doi: 10.15766/mep_2374-8265.10873 (PMC7062544; doi:10.15766/mep_2374-8265.10873)
Supplement: Supplementary file 1 — A. SP Case Development Tool Drug Screening.docx B. SP Case Development Tool Asthma.docx C. SP Case Development Tool Transfusion.docx D. SP Case Development Tool Mitochondrial.docx E. Door Notes.docx F. Learner Assessment Sheets.docx G. Debriefing Talking Points.docx H. Logistical Grid.docx I. Scenario Evaluations.docx J. OSCE Evaluation.docx K. Preevaluation for Preceptors.docx L. Postevaluation for Preceptors.docx [file mep-16-10873-s001.zip › F. Learner Assessment Sheets.docx]

Case: **Drug Screening in the Clinic** Name of Resident: Name of Preceptor:

| **Scenario Objectives** | **Milestone Competency** | **Level** | **Milestone Rubric** | **Specific Action or Behavior** | **Demonstrated** | **Comments** |
| --- | --- | --- | --- | --- | --- | --- |
| Explain the drug screening policy to patient.  Address patient’s concerns about drug screen.  Determine whether the patient will provide the urine sample today.  Make a plan for proper follow up of ADHD. | Prof 2  A sense of duty and accountability to patients, society, and the profession. | 1 | Not fully engaged and involved as a professional, which results in an observational or passive role. | Greets patient appropriately. | 🞏 Yes  🞏 No |  |
|  |  |  |  | Speaks in plain language about the problem at hand. | 🞏 Yes  🞏 No |  |
|  |  | 2 | Appreciates care providing role, but at times has difficulty seeing self as a professional, which may result in not taking appropriate responsibility. | Acknowledges that the patient’s concerns are valid. Expresses empathy. | 🞏 Yes  🞏 No |  |
|  |  | 3 | Demonstrates understanding of professional role. Fully engaged in patient care activities. Has a sense of duty. Rare lapses in behavior. | Explains reasoning for requesting drug screen in a clear and empathetic way. | 🞏 Yes  🞏 No |  |
|  |  |  |  | Reassures patient that this is private health information. | 🞏 Yes  🞏 No |  |
|  |  | 4 | Internalizes full responsibility of professional role. Fluency with patient care and professional relationships in caring for a broad range of patients and team members. | Engages in discussion about substance use and issues associated with it. | 🞏 Yes  🞏 No |  |
|  |  |  |  | Allows time for patient questions and answers them directly. | 🞏 Yes  🞏 No |  |
|  |  | 5 | Extends professional role beyond the care of patients and sees self as a professional contributing to something larger. | Gives advice to patient about how to talk to parents about the outcome of this visit. | 🞏 Yes  🞏 No |  |

Case: **Asthma in the ED** Name of Resident: Name of Preceptor:

| **Scenario Objectives** | **Milestone Competency** | **Level** | **Milestone Rubric** | **Specific Action or Behavior** | **Demonstrated** | **Comments** |
| --- | --- | --- | --- | --- | --- | --- |
| Explain to parent the recommendation to use inhaler.  Address parent’s fears of inhaler use.  Arrive at a plan of care in the ED and explain reasoning.  Arrive at a plan of care for discharge and explain reasoning. | Prof 1  Demonstrate humanism, compassion, integrity, and respect for others; based on the characteristic of an empathetic practitioner. | 1 | Interacts with patients and families in a way that is detached and not sensitive to the human needs of the patient and family. | Greets parent appropriately. | 🞏 Yes  🞏 No |  |
|  |  |  |  | Explains to parent reasons for wanting to give inhaler rather than nebulizer. | 🞏 Yes  🞏 No |  |
|  |  | 2 | Demonstrates compassion for patients in selected situations but has pattern of conduct lacking sensitivity. | Discuss with parent importance of some treatment being given to patient in the ED. | 🞏 Yes  🞏 No |  |
|  |  | 3 | Demonstrates consistent understanding of patient and family expressed needs. Responsive in demonstrating kindness and compassion. | Acknowledges parent’s concerns from previous ED visit. Expresses empathy. | 🞏 Yes  🞏 No |  |
|  |  | 4 | Goes beyond responding to expressed needs of patients and families. Is altruistic and anticipates the human needs of patients and families and works to meet those needs as part of daily practice. | Allows time for parent questions and answers them directly. | 🞏 Yes  🞏 No |  |
|  |  | 5 | Proactively advocates on behalf of individual patients, families, and groups of children in need. | Decides with parent current treatment. | 🞏 Yes  🞏 No |  |
|  |  |  |  | Decides with parent prescription upon discharge. | 🞏 Yes  🞏 No |  |

Case: **Transfusion in the Hospital** Name of Resident: Name of Preceptor:

| **Scenario Objectives** | **Milestone Competency** | **Level** | **Milestone Rubric** | **Specific Action or Behavior** | **Demonstrated** | **Comments** |
| --- | --- | --- | --- | --- | --- | --- |
| Explain to parent patient’s evolving health status.  Offer to talk to team about when transfusion is absolutely necessary.  Develop plan with parent. | Prof 6  Recognize ambiguity is part of clinical medicine and to recognize the need for and to utilize appropriate resources in dealing with uncertainty. | 1 | Demonstrates state of being overwhelmed and unsure in times of uncertainty or ambiguity. Communicates with patient/families in a limited and authoritarian manner. | Greets parent appropriately. | 🞏 Yes  🞏 No |  |
|  |  |  |  | Speaks in plain language about the problem at hand. | 🞏 Yes  🞏 No |  |
|  |  | 2 | Expresses recognition of uncertainty and pressure from not knowing. Explains situation to patient in frame most familiar to physician. | Acknowledges the parent’s concerns. Expresses empathy and openness to hearing parent’s wishes. | 🞏 Yes  🞏 No |  |
|  |  | 3 | Anticipates and focuses on uncertainty, looking for resolution by seeking additional info. Frames optimal outcome in terms of physician goals over patient/parent goals. | Acknowledges uncertainty in when to intervene. | 🞏 Yes  🞏 No |  |
|  |  |  |  | Explains to parent reasons for wanting to give transfusion and why other options aren’t available. | 🞏 Yes  🞏 No |  |
|  |  | 4 | Anticipates uncertainty is likely. Uncertainty prompts them to seek information. Balances delivering diagnosis with hopes and patient goals. | Offers to talk to team about when transfusion is absolutely necessary. | 🞏 Yes  🞏 No |  |
|  |  |  |  | Allows time for parent questions and answers them directly. | 🞏 Yes  🞏 No |  |
|  |  | 5 | Acknowledges and manages personal risk aversion or risk taking tendencies. Seeks to understand patient/family goals for help and capacity to achieve those goals. Engages in discussion with high sensitivity, emphasizing patient/family control of choices. Openly discusses the uncertainty involved in care plans. | Openly discusses strategies that take parent concerns into account and discusses anticipated outcomes with the parent. | 🞏 Yes  🞏 No |  |

Case: **Mitochondrial Disorder** Name of Resident: Name of Preceptor:

| **Scenario Objectives** | **Milestone Competency** | **Level** | **Milestone Rubric** | **Specific Action or Behavior** | **Demonstrated** | **Comments** |
| --- | --- | --- | --- | --- | --- | --- |
| Acknowledge mother’s concerns.  Explain policy of not administering medication in unlabeled container.  Support team’s decision in explanations to mother.  Describe next steps to take to get dietary plan in place. | Prof 5  Trustworthiness that makes colleagues feel secure when one is responsible for the care of patients. | 1 | Demonstrates gaps or is unaware of knowledge or skills. Demonstrates lapses in data gathering or in follow through of assigned task. May misrepresent data or omit important data, causing others to question their truthfulness. | Greets parent appropriately. | 🞏 Yes  🞏 No |  |
|  |  |  |  | Speaks in plain language about the problem at hand. | 🞏 Yes  🞏 No |  |
|  |  | 2 | Demonstrates some gaps in knowledge or skills but does not voice awareness of gaps or seek help with limitations. Lack of follow through due to inconsistency. | Acknowledges that the parent’s concerns are valid. Expresses empathy and regret about the missed dose. | 🞏 Yes  🞏 No |  |
|  |  | 3 | Demonstrates adequate level of knowledge or skills for level of responsibility. Has realistic insight into limits and data gathering is complete. | Explains previous team’s decision to follow hospital policy to parent. | 🞏 Yes  🞏 No |  |
|  |  |  |  | Reassures parent that you will follow up with nutritionist and other team members. | 🞏 Yes  🞏 No |  |
|  |  | 4 | Competent level of knowledge and skills for level of responsibility. Anticipates problems. Communication is open and includes transparent expression about uncertainty and limitation of knowledge. | Discusses what actions will now be taken to address dietary needs going forward. | 🞏 Yes  🞏 No |  |
|  |  |  |  | Allows time for parent questions and answers them directly. | 🞏 Yes  🞏 No |  |
|  |  | 5 | Competent level of knowledge and skills. Anticipates problems. Communicates uncertainty. Meticulously communicates important developments. | Offers to document new plan in medical record to assist with ongoing medical care. | 🞏 Yes  🞏 No |  |
