# Supplementary material for: Professionalism and Ethics: A Standardized Patient Observed Standardized Clinical Examination to Assess ACGME Pediatric Professionalism Milestones
Source: MedEdPORTAL. 2020 Jan 31;16:10873. doi: 10.15766/mep_2374-8265.10873 (PMC7062544; doi:10.15766/mep_2374-8265.10873)
Supplement: Supplementary file 1 — A. SP Case Development Tool Drug Screening.docx B. SP Case Development Tool Asthma.docx C. SP Case Development Tool Transfusion.docx D. SP Case Development Tool Mitochondrial.docx E. Door Notes.docx F. Learner Assessment Sheets.docx G. Debriefing Talking Points.docx H. Logistical Grid.docx I. Scenario Evaluations.docx J. OSCE Evaluation.docx K. Preevaluation for Preceptors.docx L. Postevaluation for Preceptors.docx [file mep-16-10873-s001.zip › G. Debriefing Talking Points.docx]

**Debriefing Talking Points**

**Drug Screening Case Talking Point Recommendations**

- Ask resident what options they identified to pursue with the patient, as well as the resident’s rationale for each option.
- Ask resident how they made a decision as to which option to pursue.
- Note that conversational tone should be positive, reassuring, and non-judgmental.
- This situation is an opportunity for the resident to assure the patient that they have a relationship in which it is safe to talk about sensitive topics.
- Be prepared to talk with resident about the rationale for the policy, including the extent of a resident’s duty to review clinic policies and the influence of state law regarding mandatory disclosure / breach of confidentiality (with acknowledgement that these latter 2 issues vary from state-to-state).
- Be prepared to talk with resident about the ethical considerations regarding *respect for persons* (minors) in the context of clinical care. The case requires residents to weigh the minor’s preferences, goals, responsibilities, and rights to privacy/confidentiality against duties of *beneficience* (promote patient benefit) and *non-malificence* (avoid harm). The resident should also demonstrate respect for role of the minor’s adult decision-maker (parent/guardian). In terms of the professionalism milestone, the case highlights *physican duty and accountability,* in this case duties and accountability flow to several parties (patient, parent, profession, society). Relying only on policy without further justification (i.e.- failing to comment on the avoidance of harm by reducing chance of drug-drug interactions) would be insufficient to address the milestone.

**Asthma Case Talking Point Recommendations**

- Ask resident what options they saw to pursue with the parent, as well as the resident’s rationale for each option.
- Ask resident how they made a decision as to which option to pursue.
- Review the pros and cons of nebulizer versus inhaler. Acknowledge the uncertainty and/or lack of uniformity in prescription practices. Resident should not denigrate the use of the nebulizer nor rely soley on evidence based practice to support an action.
- Identify the parent’s narrative regarding the patient’s treatment history and acknowledge parent’s confusion and frustration regarding perceived effectiveness of inhaler, especially in the context of past ICU stay.
- Discuss the ethical differences between persuading, manipulating, and coercing the parent.
- Strategize best ways to encourage inhaler use and follow up.
- Be prepared to talk with resident about the ethical considerations regarding *respect* for a minor patient’s adult decision-maker and the rights and responsibilities that attach to that role. Together with a need to demonstrate compassion, the resident should also clearly indicate *beneficience* (concern for patient well-being) and communicate that to the parent in a sensitive manner. The professionalism milestone here is *demonstration of empathy,* which asks the learner to act in ways that will not alienate the parent and will permit necessary follow up after the ER visit. Insistence that the parent is “wrong” about his/her view of efficacy and regurgitation of existing data is insufficient to address the milestone.

**Transfusion Case Talking Point Recommendations**

- Ask resident what options they saw to pursue with the parent, as well as the resident’s rationale for each option.
- Ask resident how they made a decision as to which option to pursue. Acknowledge the ambiguity/lack of certainty with respect to administration of a blood product in the context current clinical situation. Acknowledge that the prevailing approach currently is blood prduct administration, but there is variability in this setting given clinical context.
- Discuss ways to avoid medical jargon when explaining the urgency of the medical problem at hand.
- Discuss how to sensitively acknowledge to the parent that both parties have obligations, of which some are aligned and some compete.
- Review the importance of being respectful, calm, and non-judgmental about the parent and their beliefs, while acknowledging the very difficult situation as the child’s physician.
- Resident could express empathy and understanding with parents by explicitly stating the institution’s (if not the provider’s) prior experiences with faith based decisions and with JW parents in particular.
- Be prepared to talk with the resident about ethical issues related to the pediatrician’s unique role in the care of minors, where *respect for the decision-making role of the parent/guardian* may be overcome when *beneficience* (here, urgent medical needs) and *non-malificence* (avoiding harm) require physician action. Both *duty and consequentialism* are approaches that can address the benefit/risk assessment and potential mitigating factors that the resident must explore. The *relationship between law and ethical reasoning* can also be explored given the judicial attention that faith-based decision-making has received in the context of minors. The professionalism milestone here is acknowledgement and approach to treatment in the *context of uncertainty*. Threat of a court order to override the parent’s decision alone is insufficient to address this milestone.

**Mitochondrial Disorder Case Talking Point Recommendations**

- Ask resident what options they saw to pursue with the parent, as well as the resident’s rationale for each option.
- Ask resident how they made a decision as to which option to pursue.
- Discuss how to articulate regret with the parent about the missed dose of medication and the miscommunication without blaming other members of the team.
- Review the importance of listening to and allowing time for the mother to express her frustration and sense that her daughter has been wronged. Contrast the sense of being wronged with concerns about medical harm to patients.
- Advise on how to discuss an institutional policy and the basis of its legimatization.
- Discuss how ideally the resident should devise a plan that addresses the immediate problems and intends to prevent its reoccurrence.
- Be prepared to talk with the resident about *respect for persons* in the context of a distraught parent concerned about both potential harm to child and miscommunication with team. The professional milestone here is *trustworthiness* in responsible hand-off from one team member to another which permits the resident to offer reassurance about the protections within the system and yet express regret. Reliance solely on a system policy regarding outside medications would be an insufficient response to address this milestone. Additionally, simply deferring or attributing the error to the “day team” is insufficient to address this milestone.
