# Supplementary material for: Professionalism and Ethics: A Standardized Patient Observed Standardized Clinical Examination to Assess ACGME Pediatric Professionalism Milestones
Source: MedEdPORTAL. 2020 Jan 31;16:10873. doi: 10.15766/mep_2374-8265.10873 (PMC7062544; doi:10.15766/mep_2374-8265.10873)
Supplement: Supplementary file 1 — A. SP Case Development Tool Drug Screening.docx B. SP Case Development Tool Asthma.docx C. SP Case Development Tool Transfusion.docx D. SP Case Development Tool Mitochondrial.docx E. Door Notes.docx F. Learner Assessment Sheets.docx G. Debriefing Talking Points.docx H. Logistical Grid.docx I. Scenario Evaluations.docx J. OSCE Evaluation.docx K. Preevaluation for Preceptors.docx L. Postevaluation for Preceptors.docx [file mep-16-10873-s001.zip › H. Logistical Grid.docx]

| **Logistical Grid** | | | | |
| --- | --- | --- | --- | --- |
|  | Drug Screening Case | Asthma  Case | Transfusion Case | Mitochondrial Disorder Case |
| **:00** | Resident 1 | Resident 2 | Resident 3 | Resident 4 |
| **:15** | Resident 4 | Resident 1 | Resident 2 | Resident 3 |
| **:30** | Resident 3 | Resident 4 | Resident 1 | Resident 2 |
| **:45** | Resident 2 | Resident 3 | Resident 4 | Resident 1 |
