# Supplementary material for: Professionalism and Ethics: A Standardized Patient Observed Standardized Clinical Examination to Assess ACGME Pediatric Professionalism Milestones
Source: MedEdPORTAL. 2020 Jan 31;16:10873. doi: 10.15766/mep_2374-8265.10873 (PMC7062544; doi:10.15766/mep_2374-8265.10873)
Supplement: Supplementary file 1 — A. SP Case Development Tool Drug Screening.docx B. SP Case Development Tool Asthma.docx C. SP Case Development Tool Transfusion.docx D. SP Case Development Tool Mitochondrial.docx E. Door Notes.docx F. Learner Assessment Sheets.docx G. Debriefing Talking Points.docx H. Logistical Grid.docx I. Scenario Evaluations.docx J. OSCE Evaluation.docx K. Preevaluation for Preceptors.docx L. Postevaluation for Preceptors.docx [file mep-16-10873-s001.zip › I. Scenario Evaluations.docx]

Case Feedback

ID:

ADHD Drug Screening Case

Timing of the case was: 🞏 Too long 🞏 Just right 🞏 Too short

| What did you like about the case? |
| --- |
|  |

| Did you find this case realistic? Why or why not? |
| --- |
|  |

| What changes would you make to the case? |
| --- |
|  |

Additional comments/suggestions for improvement may be written below or on the back of this paper.

ID:

Asthma in the ED Case

Timing of the case was: 🞏 Too long 🞏 Just right 🞏 Too short

| What did you like about the case? |
| --- |
|  |

| Did you find this case realistic? Why or why not? |
| --- |
|  |

| What changes would you make to the case? |
| --- |
|  |

Additional comments/suggestions for improvement may be written below or on the back of this paper.

ID:

Transfusion Case

Timing of the case was: 🞏 Too long 🞏 Just right 🞏 Too short

| What did you like about the case? |
| --- |
|  |

| Did you find this case realistic? Why or why not? |
| --- |
|  |

| What changes would you make to the case? |
| --- |
|  |

Additional comments/suggestions for improvement may be written below or on the back of this paper.

ID:

Mitochondrial Disorder Case

Timing of the case was: 🞏 Too long 🞏 Just right 🞏 Too short

| What did you like about the case? |
| --- |
|  |

| Did you find this case realistic? Why or why not? |
| --- |
|  |

| What changes would you make to the case? |
| --- |
|  |

Additional comments/suggestions for improvement may be written below or on the back of this paper.
