# Supplementary material for: Professionalism and Ethics: A Standardized Patient Observed Standardized Clinical Examination to Assess ACGME Pediatric Professionalism Milestones
Source: MedEdPORTAL. 2020 Jan 31;16:10873. doi: 10.15766/mep_2374-8265.10873 (PMC7062544; doi:10.15766/mep_2374-8265.10873)
Supplement: Supplementary file 1 — A. SP Case Development Tool Drug Screening.docx B. SP Case Development Tool Asthma.docx C. SP Case Development Tool Transfusion.docx D. SP Case Development Tool Mitochondrial.docx E. Door Notes.docx F. Learner Assessment Sheets.docx G. Debriefing Talking Points.docx H. Logistical Grid.docx I. Scenario Evaluations.docx J. OSCE Evaluation.docx K. Preevaluation for Preceptors.docx L. Postevaluation for Preceptors.docx [file mep-16-10873-s001.zip › J. OSCE Evaluation.docx]

id Resident Post Survey

**Please rank the cases in terms of:**

| **How realistic they are from 1 to 4**  *(1 = most realistic and 4 = least realistic)* | **How much you liked them from 1 to 4**  *(1 = most liked and 4 = least liked)* |
| --- | --- |
| ADHD Drug Screen Case | ADHD Drug Screen Case |
| Asthma in the ER Case | Asthma in the ER Case |
| Jehovah’s Witness Case | Jehovah’s Witness Case |
| Mitochondrial Disorder Case | Mitochondrial Disorder Case |

**Please rank the cases in terms of:**

| **How challenging they are from 1 to 4**  *(1 = most challenging and 4 = least challenging)* | **How well they assessed professionalism from 1 to 4**  *(1 = best at assessing and 4 = worst at assessing)* |
| --- | --- |
| ADHD Drug Screen Case | ADHD Drug Screen Case |
| Asthma in the ER Case | Asthma in the ER Case |
| Jehovah’s Witness Case | Jehovah’s Witness Case |
| Mitochondrial Disorder Case | Mitochondrial Disorder Case |

| **For the case you ranked first in terms of professionalism, why do you think this case is best at measuring professionalism?** |
| --- |
|  |

| **Which aspects of these OSCEs did you find the most useful?** |
| --- |
|  |
