# Supplementary material for: Professionalism and Ethics: A Standardized Patient Observed Standardized Clinical Examination to Assess ACGME Pediatric Professionalism Milestones
Source: MedEdPORTAL. 2020 Jan 31;16:10873. doi: 10.15766/mep_2374-8265.10873 (PMC7062544; doi:10.15766/mep_2374-8265.10873)
Supplement: Supplementary file 1 — A. SP Case Development Tool Drug Screening.docx B. SP Case Development Tool Asthma.docx C. SP Case Development Tool Transfusion.docx D. SP Case Development Tool Mitochondrial.docx E. Door Notes.docx F. Learner Assessment Sheets.docx G. Debriefing Talking Points.docx H. Logistical Grid.docx I. Scenario Evaluations.docx J. OSCE Evaluation.docx K. Preevaluation for Preceptors.docx L. Postevaluation for Preceptors.docx [file mep-16-10873-s001.zip › K. Preevaluation for Preceptors.docx]

id Pre-Evaluation Survey for Preceptors

| Please provide information about yourself and your residency program |
| --- |
| Your medical specialty: 🞏 Medicine/Pediatrics 🞏 Pediatrics 🞏 Other ______________________________  Where did you do your residency? ______________________________    How much did your residency program value professionalism? 🞏 Not at all 🞏 Some 🞏 Quite a bit  How many years have you been in practice (beyond residency)? 🞏 0-5 years 🞏 6-10 years 🞏 over 10 years |

| How comfortable are you with assessing and providing feedback using these professional milestones? | very somewhat somewhat very  uncomfortable uncomfortable neutral comfortable comfortable |
| --- | --- |
| PROF1: Humanism, compassion, integrity, and respect for others; based on the characteristics of an empathetic practitioner | 🞏 ---------------------- 🞏 ---------------------- 🞏 ---------------------- 🞏 ---------------------- 🞏 |
| PROF2: Professionalization; a sense of duty and accountability to patients, society, and the profession | 🞏 ---------------------- 🞏 ---------------------- 🞏 ---------------------- 🞏 ---------------------- 🞏 |
| PROF3: Professional conduct; high standards of ethical behavior which includes maintaining appropriate professional boundaries | 🞏 ---------------------- 🞏 ---------------------- 🞏 ---------------------- 🞏 ---------------------- 🞏 |
| PROF4: Self-awareness of one’s own knowledge, skill, and emotional limitations that leads to appropriate help-seeking behaviors | 🞏 ---------------------- 🞏 ---------------------- 🞏 ---------------------- 🞏 ---------------------- 🞏 |
| PROF5: Trustworthiness that makes colleagues feel secure when one is responsible for the care of patients | 🞏 ---------------------- 🞏 ---------------------- 🞏 ---------------------- 🞏 ---------------------- 🞏 |
| PROF6: The capacity to accept that ambiguity is part of clinical medicine and to recognize the need for and to utilize appropriate resources in dealing with uncertainty | 🞏 ---------------------- 🞏 ---------------------- 🞏 ---------------------- 🞏 ---------------------- 🞏 |

|  | very somewhat somewhat very  uncomfortable uncomfortable neutral comfortable comfortable |
| --- | --- |
| How comfortable are you giving residents feedback regarding the ethical issues that appear in the professionalism milestones? | 🞏 ---------------------- 🞏 ---------------------- 🞏 ---------------------- 🞏 ---------------------- 🞏 |
