# Supplementary material for: Professionalism and Ethics: A Standardized Patient Observed Standardized Clinical Examination to Assess ACGME Pediatric Professionalism Milestones
Source: MedEdPORTAL. 2020 Jan 31;16:10873. doi: 10.15766/mep_2374-8265.10873 (PMC7062544; doi:10.15766/mep_2374-8265.10873)
Supplement: Supplementary file 1 — A. SP Case Development Tool Drug Screening.docx B. SP Case Development Tool Asthma.docx C. SP Case Development Tool Transfusion.docx D. SP Case Development Tool Mitochondrial.docx E. Door Notes.docx F. Learner Assessment Sheets.docx G. Debriefing Talking Points.docx H. Logistical Grid.docx I. Scenario Evaluations.docx J. OSCE Evaluation.docx K. Preevaluation for Preceptors.docx L. Postevaluation for Preceptors.docx [file mep-16-10873-s001.zip › L. Postevaluation for Preceptors.docx]

id Post-Evaluation Survey for Preceptors

|  | poor fair average good excellent |
| --- | --- |
| Please rate the OSCE overall | 🞏 ------------------- 🞏 ------------------- 🞏 ------------------- 🞏 ------------------- 🞏 |

| Please offer your opinion | strongly somewhat somewhat strongly  disagree disagree neutral agree agree |
| --- | --- |
| It is useful to have a milestone simulation assessment program tailored to the professionalism milestones | 🞏 ------------------- 🞏 ------------------- 🞏 ------------------- 🞏 ------------------- 🞏 |
| It was useful to examine the ethical issues as contained in the professionalism milestones | 🞏 ------------------- 🞏 ------------------- 🞏 ------------------- 🞏 ------------------- 🞏 |
| I felt comfortable assessing and providing feedback on the professional milestones | 🞏 ------------------- 🞏 ------------------- 🞏 ------------------- 🞏 ------------------- 🞏 |
| The cases were appropriate for the competencies being measured | 🞏 ------------------- 🞏 ------------------- 🞏 ------------------- 🞏 ------------------- 🞏 |
| The descriptions in the grading rubrics made competency scores easy to assess | 🞏 ------------------- 🞏 ------------------- 🞏 ------------------- 🞏 ------------------- 🞏 |
| The OSCE was run efficiently | 🞏 ------------------- 🞏 ------------------- 🞏 ------------------- 🞏 ------------------- 🞏 |
| I felt my time was well spent while participating | 🞏 ------------------- 🞏 ------------------- 🞏 ------------------- 🞏 ------------------- 🞏 |
| This OSCE improved my ability to assess the professionalism milestones | 🞏 ------------------- 🞏 ------------------- 🞏 ------------------- 🞏 ------------------- 🞏 |

How many times do you recommend assessing the professionalism milestones with simulation during training?

🞏 1x/year 🞏 2x/year 🞏 2x in all of residency

| Which topics or aspects of the OSCE did you find most interesting or useful? |
| --- |
|  |

| How has the OSCE affected how you might provide feedback on the professional milestones? |
| --- |
|  |

| What didn’t we cover today that would have been useful? |
| --- |
|  |
